# Long-lived Humans Have a Unique Plasma Sphingolipidome

**DOI:** 10.1093/gerona/glab360

**Published:** 2021-12-05

**Authors:** Irene Pradas, Mariona Jové, Kevin Huynh, Marta Ingles, Consuelo Borras, Natalia Mota-Martorell, Jose Daniel Galo-Licona, Josep Puig, Jose Viña, Peter J Meikle, Reinald Pamplona

**Affiliations:** 1 Department of Experimental Medicine, University of Lleida-Lleida Biomedical Research Institute (UdL-IRBLleida), Lleida, Catalonia, Spain; 2 Baker Heart and Diabetes Institute, Melbourne, VIC, Australia; 3 Department of Physiology, University of Valencia, Valencia, Spain; 4 Girona Biomedical Research Institute (IDIBGI), Hospital Universitari Dr Josep Trueta, Girona, Catalonia, Spain

**Keywords:** Aging, Centenarians, Ceramides, Glycosphingolipids, Mass spectrometry

## Abstract

A species-specific lipidome profile is an inherent feature linked to longevity in the animal kingdom. However, there is a lack of lipidomic studies on human longevity. Here, we use mass spectrometry-based lipidomics to detect and quantify 151 sphingolipid molecular species and use these to define a phenotype of healthy humans with exceptional life span. Our results demonstrate that this profile specifically comprises a higher content of complex glycosphingolipids (hexosylceramides and gangliosides), and lower levels of ceramide species from the de novo pathway, sphingomyelin and sulfatide; while for ceramide-derived signaling compounds, their content remains unchanged. Our findings suggest that structural glycosphingolipids may be more relevant to achieve the centenarian condition than signaling sphingolipids.

Maximum life span is a species-specific trait being about 115–120 years in humans (1,2). Centenarians are considered an exceptional human model of healthy aging and extreme longevity (3,4). Available evidence suggests a link between maximum life span and lipids (5). The findings from several comparative studies (using vertebrates, invertebrates, and exceptionally long-lived animal species) demonstrate that the cell membrane lipid profile has been an optimized evolutionary adaption (5). This adaption is expressed as the longer the maximum life span, the higher the lipid resistance to oxidation (5–7). Reinforcing the hypothesis, a phylogenomic approach showed that genes involved in lipid metabolism have undergone an increased selective pressure in long-lived species (8). As an extension of these findings, recent mass spectrometry-based lipidomics studies confirm that the lipidome is also species specific and is associated with species’ longevity (9,10). Furthermore, some studies have demonstrated the presence of favorable lipid profiles in offspring of centenarians suggestive of genotypic adaptations in lipid metabolism associated with human life span (11–13). All these observations point to lipids as a key target to study the molecular adaptive mechanisms underlying differences in life span.

A major category of lipids present in all eukaryotic cells is sphingolipids (14). Sphingolipids are a highly conserved lipid component of cell membranes with an inherent predisposition to lateral segregation within lipid bilayer leading to the formation of lipid rafts (15). The metabolism of sphingolipids is a complex network of pathways where ceramide plays the role of a central metabolic hub (16). Ceramide can be synthesized by 3 main pathways: the de novo pathway, hydrolysis of complex sphingolipids, and the salvage pathway. The result is a wide diversity of lipid species with structural and bioactive/messenger functions. Structural sphingolipids include sphingomyelins (SM), and glycosphingolipids (including hexosylceramides (HexCer), gangliosides (GM), and sulfatides), whereas bioactive sphingolipids are represented by sphingosine (Sph), sphingosine-1-phosphate (Sph-1-P), ceramide-1-phosphate (Cer-1-P), dihydroceramide (dhCer), and ceramide (Cer) (14). Sphingolipids regulate membrane physiology and related functions (fluidity, organization, geometry, lipid-protein and protein-protein interactions, membrane trafficking, clustering of plasma membrane receptors and ion channels, signal transduction, and cytoskeletal organization), cell biology (oxidative stress, apoptosis, cell survival, autophagy, endosomes and endocytosis, cell cycle, proliferation, migration, and senescence), and complex physiological systems (development, aging, and life span); and they have been involved in several pathological conditions such as inflammation, metabolic and cardiovascular diseases, neurodegeneration, and cancer (16–19). In relation to the link between sphingolipids and life span, evidence in model organisms such as yeast and fly suggest that deletions or mutations of genes affecting ceramide biosynthesis or metabolism are able to significantly modify mean and maximum life span (20–24).

Although systems biology-based approaches allow a comprehensive molecular characterization of complex biological systems, to date, no targeted lipidomic analysis investigating differences in the plasma sphingolipidome of exceptionally long-lived humans have been reported. To this end, we have designed a study that represents the most detailed plasma sphingolipidomic analysis associated with human longevity to detect and quantify a panel of sphingolipids including 151 molecular species: 7 sphingosines (Sph), 56 ceramides (Cer), 44 sphingomyelins (SM), 30 hexosylceramides (HexCer, Hex2Cer, Hex3Cer), 8 gangliosides (GM), and 6 sulfatides. The plasma sphingolipid profile was determined using a LC-MS/MS platform to systematically define specific phenotypic patterns associated with genotypes of human extreme longevity. We identified a particular sphingolipid signature related to the condition of extreme longevity, allowing the identification of potential mechanisms and biomarkers of healthy aging.

## Material and Methods

### Chemicals

Lipid internal standards (ISTD) included species within the categories of glycerophospholipids, sphingolipids, and glycerolipids. Sph(d17:1), SM(30:1), dhCer(d18:0/8:0), Cer(d18:1/17:0), and sulfatide(d18:1/12:0) were obtained from Avanti (Alabaster, AL). Glucosylceramide (GluCer(d18:1/16:0) (d3)), lactosylceramide (LacCer(d18:1/16:0) (d3)) and trihexosylceramide (Hex3Cer(d18:1/17:0) were purchased from Matreya (Pleasant Gap, PA). The solvents 1-butanol, methanol, and chloroform were HPLC-grade and purchased from Merck KGaA (Darmstadt, Germany).

### Study Population

Potential healthy subjects were selected from the population data system of the 11th Health Department of the Valencian Community (Valencia, Spain), which is composed of 29 towns (240 000 inhabitants). The inclusion criteria were to live in the 11th Health Department for at least the last 6 years and to sign the informed consent. The exclusion criterion was to be terminally ill for any reason. We found 25 (6 males/19 females) centenarians (age 100.8 ± 1.1 years); 22 (7 males/15 females) randomly recruited aged subjects (age 76.4 ± 0.5 years), and 21 (7 males/14 females) adult individuals (age 27.9 ± 1.4 years). All experimental procedures were approved by the Committee for Ethics in Clinical Research of the Hospital de la Ribera (Alzira, Valencia, Spain). All subjects or their relatives were fully informed of the aims and scope of the research and signed an informed consent.

The baseline characteristics of the study groups including basic lipid biochemical determinations reveal no significant differences for high-density lipoprotein (HDL)-cholesterol, very-low-density lipoprotein (VLDL)-cholesterol, free cholesterol, and plasma triacylglycerides among groups; in contrast, total-cholesterol and low-density lipoprotein (LDL)-cholesterol showed significantly increased levels in the aged group compared to adults and centenarians (25).

### Targeted Lipidomics

#### Blood collection and plasma isolation

Blood samples were obtained by venipuncture in the morning (between 7 and 8 a.m.) after fasting overnight (8–10 hours) and collected in one Vacutainer CPT (Cell Preparation Tube; BD, Franklin Lakes, NJ) containing sodium heparin as the anticoagulant. Plasma fraction were collected after blood sample centrifugation, and immediately frozen in liquid nitrogen, and transferred before 4 hours to a −80°C freezer for storage, to be used later for lipidomic analyses.

#### Sample preparation and lipid extraction

Plasma samples from the subjects were randomized prior to lipid extraction. Quality control plasma samples were included at a ratio of 1:10. Samples were thawed and 1 µL of the antioxidant butylhydroxytoluene (100 mM in ethanol) per 1 mL of plasma was added. To each plasma sample (10 µL), a mixture of internal standards in chloroform:methanol (1:1) was added. Lipids were extracted in a single-phase chloroform:methanol (2:1) procedure as described previously (26). Lipid standards stock solutions were prepared by dissolving lipid standards (Supplementary Table 1) in chloroform:methanol (1:1,v/v) at 100 pmol except for cholesterol that was 10 000 pmol, cholesteryl ester 1 000 pmol, and sphingomyelin and diacylglycerol that were at 200 pmol. Finally, for the dihydroceramides and hexosylceramides, the working solutions were at 50 pmol. The mixture was mixed for 10 minutes on a rotary mixer, sonicated in a water bath (18–24°C) for 30 minutes, left to stand on the bench for 20 minutes and then centrifuged at 16 000 × *g* at 20°C for 10 minutes. The supernatant was transferred to a 96-well plate and dried under a stream of nitrogen gas at 40°C. Samples were reconstituted with 50 μL H_2_O-saturated 1-butanol and sonicated for 10 minutes. Then, 50 μL of 10 mM ammonium formate in methanol was added. The extract was centrifuged at 1 700 × *g* at 20°C for 5 minutes. The recovery efficiencies of the lipid extraction method for each lipid subclass were previously published in other work (27). Finally, supernatant was transferred into a 0.2 mL glass insert with Teflon insert cap for analysis by LC ESI-MS/MS.

### Lipidomic analysis

Lipidomic analysis was performed by an LC-ESI-QQQ MS/MS model 6490 from Agilent Technologies (Melbourne, Australia). Full methodology is described by Huynh et al. (26). Briefly, 1 μL of lipid extract was applied onto ZORBAX eclipse plus C18 column, 2.1 × 100 mm 1.8 μm (Agilent Technologies), heated to 60°C and the auto-sampler regulated to 25°C. Flow rate was 400 μL/min with solvent A composed of 10 mM ammonium formate in acetonitrile-water-isopropanol (50:30:20, v/v) and solvent B composed of 10 mM ammonium formate in acetonitrile-water-isopropanol (9:1:90, v/v). The gradient started at 10% of mobile phase B, reached 100% B in 11 minutes and held for 1 minute. Finally, the system was switched back to 10% of mobile phase B and was equilibrated for 3 minutes. Data were collected in the multiple reaction monitoring scan type and the capillary voltage was set at 3 500 V. Positive polarity of electrospray ionization was set using N_2_ at 20 psi as nebulizer gas (17 L/min, 150°C) and the sheath gas parameters were flow at 10 L/min and temperature at 200°C. For all the lipid species, the cell accelerator voltage was 5 volts, fragmentor was 380 volts. Conditions for tandem mass spectrometry quantification of major lipid species internal standards are detailed in Supplementary Table 1.

#### Data analysis

The MassHunter Data Analysis Software (Agilent Technologies) was used to collect the results and the software MassHunter Quantitative Analysis (Agilent Technologies) was used to quantify every lipid species in the samples. Concentrations were obtained in nmol/mL. Multivariate statistics (hierarchical clustering and principal component analysis) were done using Metaboanalyst software and multivariate linear regression adjusting for sex and clinical lipids (cholesterol and triacylglycerides) with all *p*-values were corrected for multiple comparisons using the Benjamini–Hochberg approach. *p* values of less than .05 were considered significant. Analyses were performed using R statistical software, version 3.4.4.

## Results

**Figure 1.**
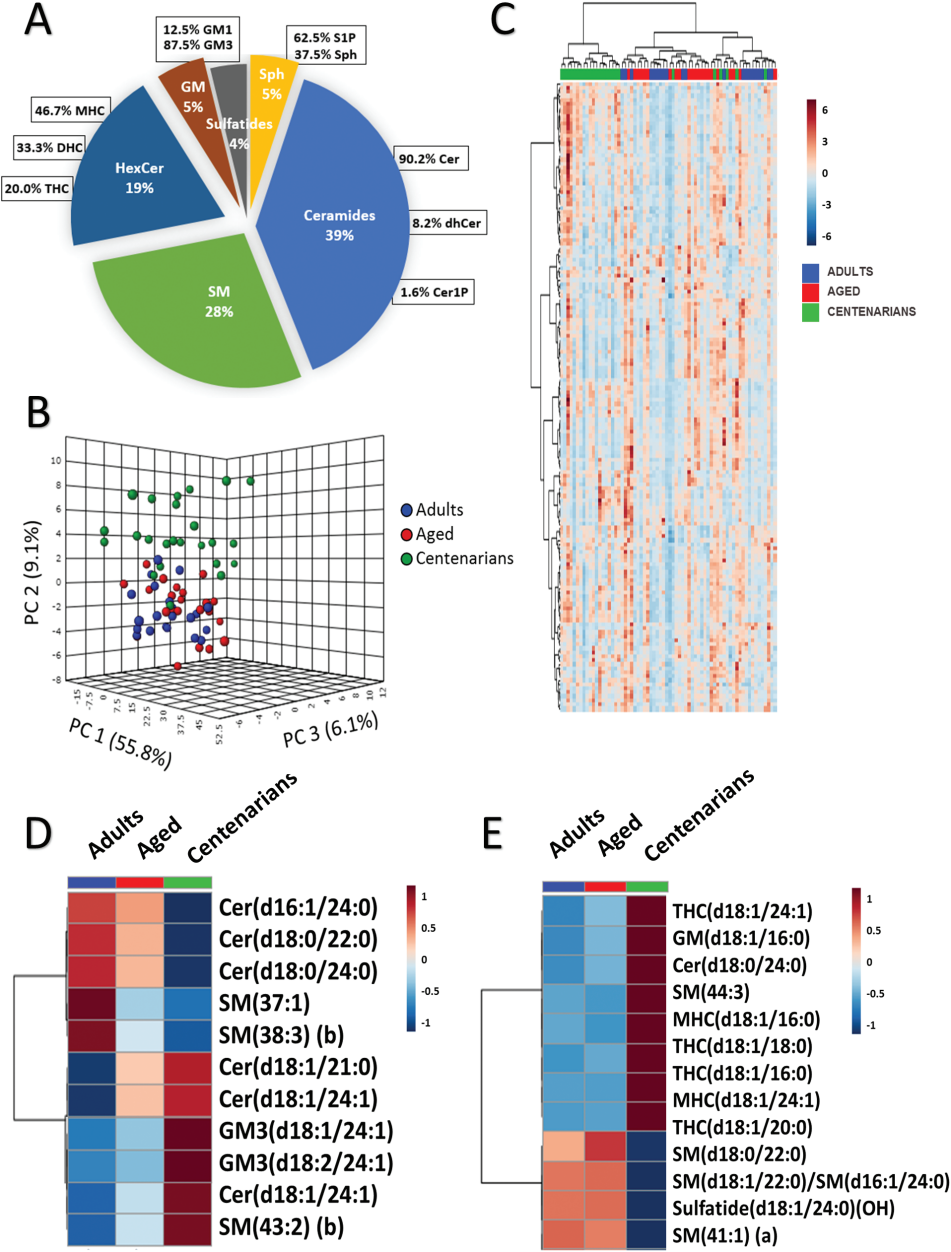
Signaling lipidomic signature of aging and longevity in human plasma. (**A**) Graphical representation of all the analyzed lipid species clustered by subclasses. (**B**) Principal component analysis of the centenarians (green), aged subjects (red) and adults (blue) based on the 151 sphingolipid species analyzed. (**C**) Heat map representation of hierarchical clustering of 151 lipid species analyzed. Each line of this graphic represents a sphingolipid species, colored by its abundance intensity normalized across the samples. The scale from -7 (blue) to 7 (red) represents this normalized abundance in arbitrary units. (**D**) Heat map with a set of metabolites that decrease or increase with age. (**E**) Heat map with a set of metabolites that decrease or increase in centenarians only. Species separated chromatographically but incompletely characterized were labeled with (a) or (b), where (a) and (b) represent the elution order. MHC, Hex1Cer; THC, Hex3Cer.

In the present study, 151 sphingolipid species in human plasma were measured. This broad panel of lipids includes the following: 7 sphingosines (Sph) with 4 of them conjugated with phosphate (S1P), 56 ceramides (Cer) including one ceramide-1-phosphate (Cer1P) and 5 dihydroceramides (dhCer), 44 sphingomyelins (SMs), 14 monohexosylceramides (HexCer), 10 dihexosylceramides (Hex2Cer), 6 trihexosylceramides (Hex3Cer), 8 gangliosides (GM) one of them GM1 and the rest GM3, and 6 sulfatides (Figure 1A). The baseline characteristics of the study groups including basic lipid biochemical determinations were previously published in Pradas et al. (25). No significant differences were observed for HDL-cholesterol, VLDL-cholesterol, free cholesterol, and plasma triacylglycerides among groups; in contrast, total-cholesterol and LDL-cholesterol showed significantly increased levels in the aged group compared to adults and centenarians (25).

In order to evaluate the contribution of these sphingolipid species to plasma variability, we have analyzed the lipid profile from the experimental groups (adults, aged, and centenarians). Principal Component Analysis (Figure 1B) showed 55.8% of the variability explained by the component 1 that was independent of the experimental groups. However, centenarian subjects were clustered apart from the other groups in the second component, which explained 9.1% of the variability across samples. When a hierarchical clustering algorithm was performed the resulting heat map showed a different pattern in centenarians although experimental groups did not cluster completely, with some adults and aged subjects mixed (Figure 1C).

Univariate statistical analysis revealed 2 distinct metabolic patterns among the statistically significant sphingolipids (Figure 1D and E). The first pattern was comprised of a set of lipid species that increase or decrease with age, likely representing the fingerprint of the aging process, being decreased a couple of dhCer and SM and being increased Cer, SM(43:2) (b) and a couple of GM3 lipid species (Figure 1D). The amount of sphingolipid species that correlated with aging represents a 7% of the 151 lipid species detected (Supplementary Table 2). On the other hand, the second pattern was comprised by a set of lipid species that increase or decrease in a specific way in centenarians, sharing the adults and aged the same abundancy. Several HexCer were increased in centenarians, while sulfatide(d18:1/24:0(OH)) and a few SM were decreased (Figure 1E). Therefore, plasma levels of different species of structural and signaling sphingolipid species are correlated with the aging process while some of them with structural functions show specific levels in the centenarian experimental group compared to adults and aged. Supplementary Table 2 offers information about the plasma concentration for all the sphingolipid species analyzed in adults, aged, and centenarians.

**Figure 2.**
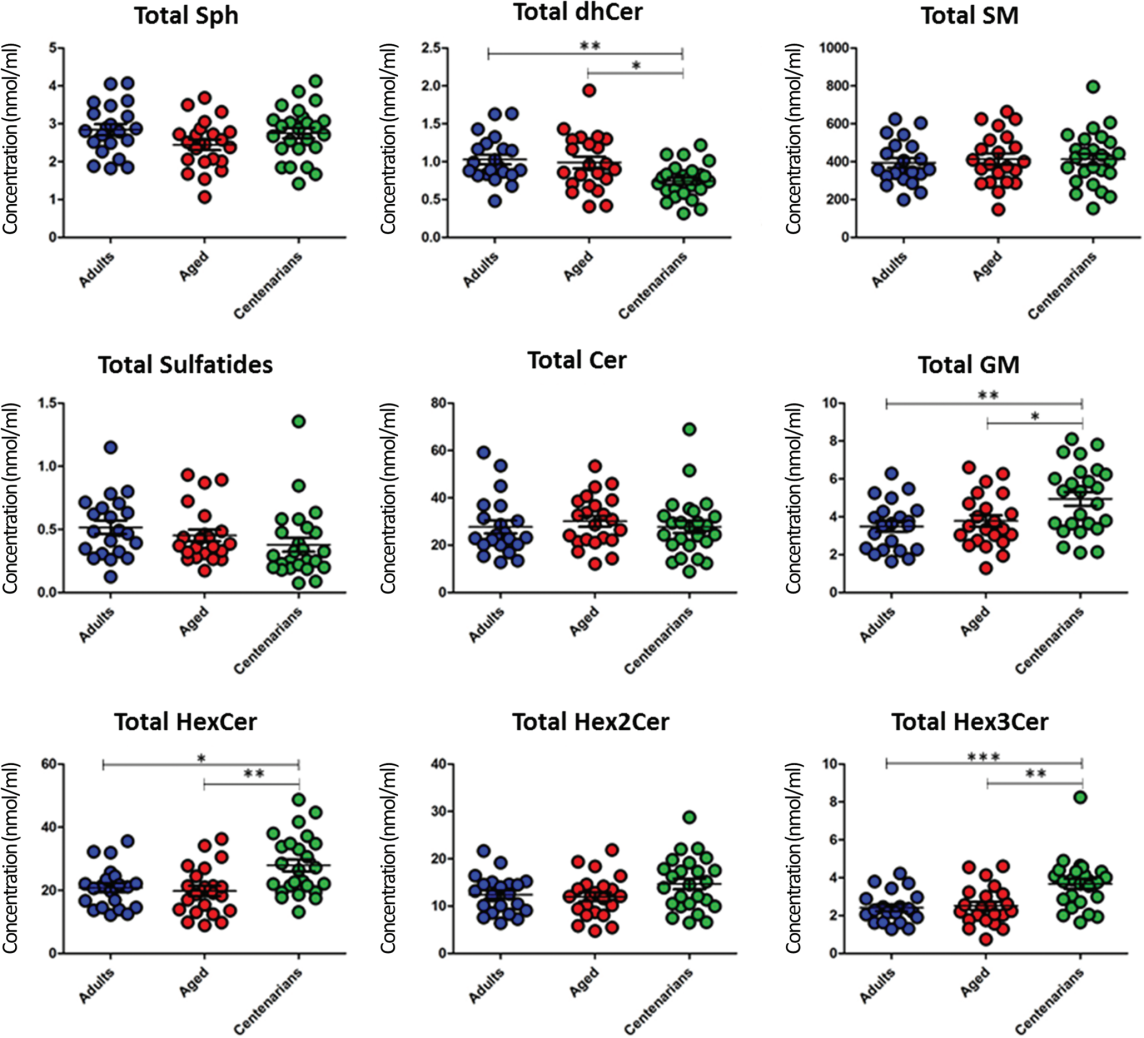
Graphical representation of sphingolipid species concentrations in each experimental group (adults, aged, and centenarians) clustered by lipid subclasses. The expressed concentrations in nmol/mL of plasma are the experimental group mean of the sum of the all the molecular species within that class or subclass of each individual. *p*-values obtained by 1-way ANOVA post hoc Tukey’s *p < .05; **p < 0.01, and ***p < .001.

Furthermore, we tested whether general differences according to the analyzed lipid subclasses existed among the experimental groups (adults, aged, and centenarians) (Figure 2). The results show that total levels of the Sph, Cer, SM, Hex2Cer, and sulfatide subclasses remained unaltered among the different groups. The subclass dhCer was decreased in centenarians with respect to adults (*p* < .01) and aged subjects (*p* < .05) (Figure 2). Glycosphingolipid subclasses showed differences in HexCer, statistically higher in centenarians with respect to adults (*p* < .05) and aged subjects (*p* < .01) and Hex3Cer, statistically higher in centenarians with respect to adults (*p* < .001) and aged subjects (*p* < .01) (Figure 2). Gangliosides showed an increased level in centenarians with respect to the adults (*p* < .01) and aged subjects (*p* < .05) (Figure 2).

**Figure 3.**
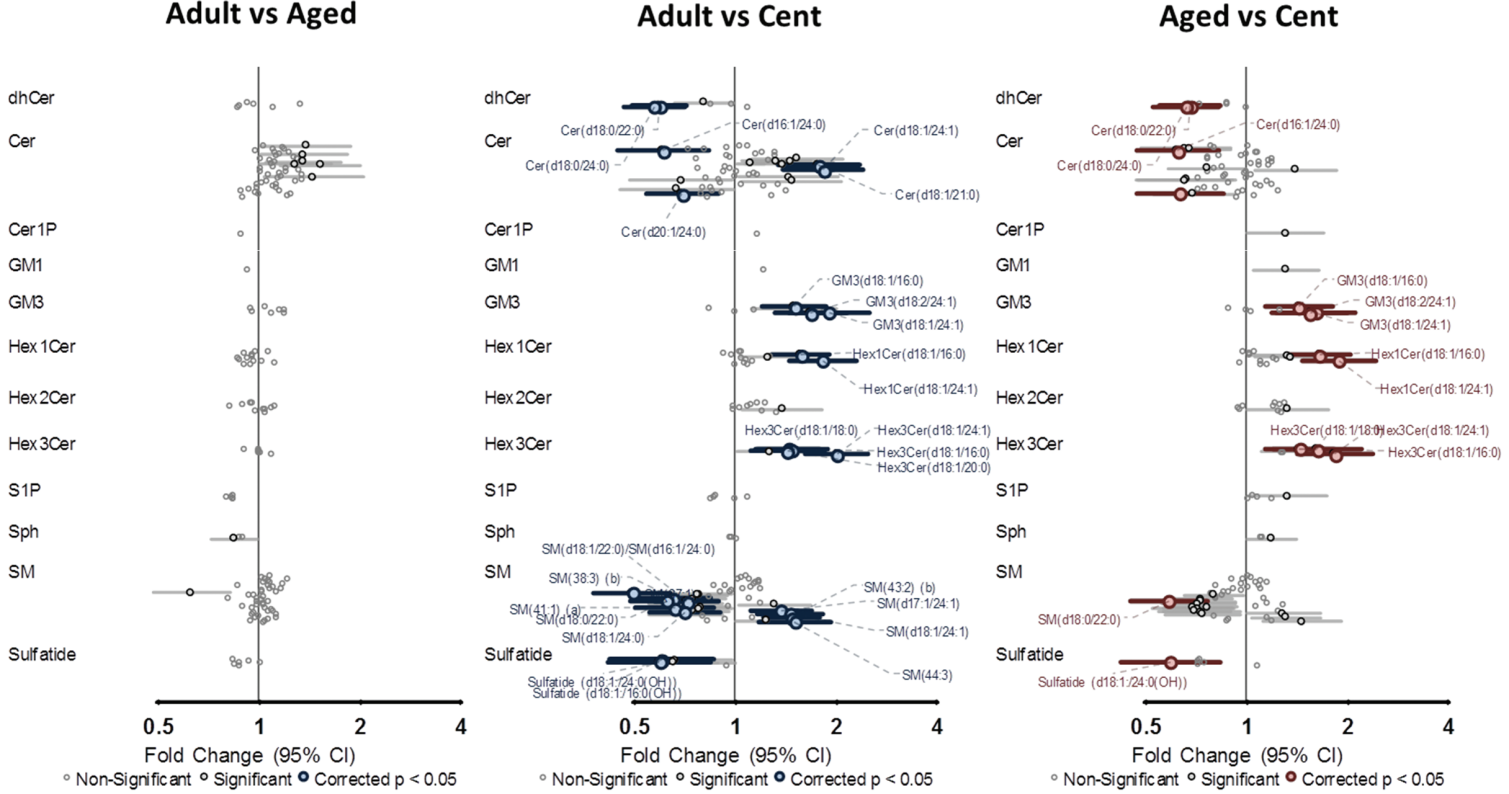
Multinomial linear regression model examining the association between sphingolipid species and the experimental groups (adults, aged, and centenarians). Highlighted can be seen the lipid species significantly different between the experimental groups.

Multinomial regression models were performed with the centenarian group as reference in order to find associations and differential sphingolipid species. Supplementary Table 3 shows multinomial linear regression models examining the association between sphingolipid species and the experimental groups (adults, aged, and centenarians). Thus, 57 of the 151 detected lipid species of the sphingolipidome (37.7%) were statistically different in adults or aged compared to centenarians. Among them, 30 (19.8%) lipid species were different between centenarians and the other 2 groups, 9 (5.9%) just between centenarians and aged, and 18 (11.9%) just between centenarians and adults (Figure 3). In particular, when adults are compared to centenarians, the differential molecular species belong 39.6% to SM followed by Cer (25%); HexCer (16.7%); GM (10.4%); and finally, dhCer and sulfatides (4.2% each of the subclasses). When the aged group is compared to centenarians, the affected molecules were 38.5% SM, 23.1% HexCer, 15.4% Cer, 10.3% GM, 5.1% dhCer and 2.6% of Cer1P, Sph, and sulfatides. Therefore, both groups (adults and aged) present a similar proportion among the differential lipid species compared to centenarians for SM, dhCer, Cer, and acidic glycosphingolipid species, such as gangliosides and sulfatides. In this last subclass, 2 different trends can be appreciated being all the differential GM species increased in centenarians and oppositely all the sulfatides decreased. Among the HexCer subclass, a couple of HexCer species were highly increased in centenarians, no changes were observed for Hex2Cer species and the main differences were ascribed to Hex3Cer lipid species being all of them increased.

**Figure 4.**
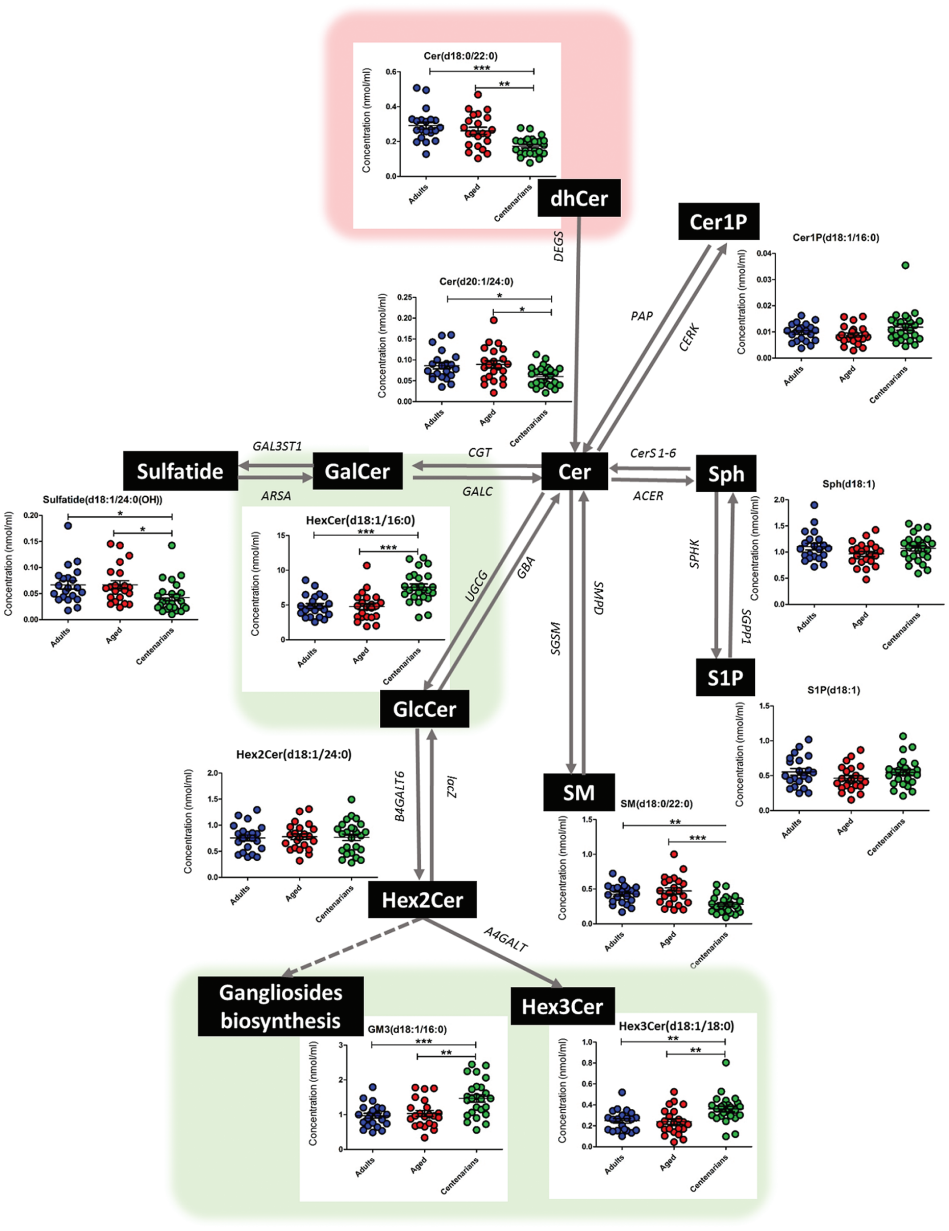
Biosynthesis pathways of main sphingolipid species and its status in adults (blue), aged (red), and centenarians (green). DEGS, sphingolipid delta-4-desaturase; PAP, phosphatidate phosphatase; CERK, ceramide kinase; ACER, alkaline ceramidase; CerS 1-6, ceramide synthetase; SPHK, sphingosine kinase; SGPP1, sphingosine-1-phosphate phosphatase; CGT, ceramide galactosyltransferase; GALC, galactosylceramidase; GAL3ST1, galactosylceramide sulfotransferase; ARSA, arylsulfatase A; SGMS, sphingomyelin synthase; SMPD, sphingomyelin phosphodiesterase; UGCG; ceramide glucosyltransferase; GBA, glucosylceramidase; B4GALT6, beta-1,4-galactosyltransferase 6; lacZ, beta-galatosidase; A4GALT, Lactosylceramide 4-alpha-galactosyltransferase.

Focusing on the analyzed signaling sphingolipid species, specific differences in centenarians were observed in almost all of the subclasses except for Cer1P, Sph, S1P, and Hex2Cer (Figure 3). Regarding dhCer, the trend for all the analyzed species was to be decreased in centenarians. Both subclasses, SM and Cer, showed a lipid-specific regulation without sharing a common pattern in centenarians respect to adults and aged subjects (Figure 3). Oppositely, as it has been presented a centenarian-specific pattern was observed for structural sphingolipid species HexCer, Hex3Cer, and GM being increased in centenarians while sulfatides were decreased. Representative lipid species have been chosen for each subclass and introduced into the scheme of the biosynthesis pathway helping to unravel the status of the metabolic pathway in each experimental group (Figure 4).

Finally, there is a significant difference in the concentration between ceramides and hexosylceramides esterified to C24:0 and those esterified to C24:1. To look further into this, we calculated the ratio of significant ceramides and hexosylceramides esterified to C24:0 to ceramides and hexosylceramides esterified to C24:1 (Supplementary Table 4). As a result, centenarians had a lower C24:0:C24:1 ratio across all ceramides and hexosylceramides species when compared with both experimental groups.

## Discussion

Although their wide presence and abundance in living organisms and their relevance to human health, the detection and quantification of sphingolipids and their association with complex processes such as aging and longevity has been performed in a reduced number of studies, and no conclusive results have been obtained yet. Nevertheless, the findings obtained from lipidomic studies in animal models such as the yeast Saccharomyces *cerevisiae* (28), the worm Caenorhabditis *elegans* (29), the exceptional long-lived naked mole-rat (*Heterocephalus glaber*) (30), and mammals differing in maximum longevity (9,10) suggest an association between sphingolipid content and animal longevity. Specifically, low sphingolipid content, particularly for sulfatides, ceramides, and glycosphingolipids, seems to be a trait shared by long-lived species. Additional studies based on pharmacological or genetic interventions inducing decreased sphingolipid synthesis also result in increased animal life span (28,29,31).

Lipidomic studies on human aging and longevity are very limited in number and the results are not conclusive, basically due to the reduced number of sphingolipid species analyzed, and the fact that these molecular species are restricted to sphingomyelins (13,32–36). Our results demonstrate a specific sphingolipidome in centenarians. This sphingolipid signature can globally be ascribed to a lower concentration of molecular species involved in ceramide biosynthesis, a lower content of sulfatides and sphingomyelins, and greater content of hexosylceramides and gangliosides; no changes were observed for ceramide-derived molecular species with a signaling role. So, our findings suggest that structural glycosphingolipids instead of sphingolipids with signaling roles are more determinants in the definition of a centenarian phenotype.

Assuming that the plasma lipidome integrates lipid pathways operating in the different cell types of the human body, the plasma sphingolipidome may be interpreted as the expression of what is happening in cell membrane microdomains where sphingolipids are predominantly located. In this scenario, and in order to explain the meaning of the specific sphingolipid profile observed in centenarians, it is hypothesized that the down-regulation of ceramides, sphingomyelins, and sulfatides synthesis and up-regulation of glycosphingolipids content is a phenotype which preserves the functional properties of cell membrane, as well as signal transduction pathways and cellular processes favoring a high longevity. Further studies are, however, needed to develop a more detailed view with a special attention to the analysis of the lipidomic profiles at tissue level as, to the best of our knowledge, no data are currently available on centenarians.

Our results also showed how ceramides and hexosylceramides esterification to C24:1 increases relative to the C24:0 species in centenarians, suggesting a differential regulation of the desaturase scd1 which should be investigated with further studies. However, a recent work in centenarians (13) reported no differences in plasma monounsaturated fatty acid content of total lipids in centenarians compared to both adults and aged individuals. So, although changes in monounsaturated content of other specific lipid categories or classes cannot be discarded, these results suggest that instead of changes in scd1 activity, are changes in the biosynthesis of specific sphingolipids or even in their remodeling potentially more relevant. In any case, the significance of this finding is interesting because expresses an enrichment in monounsaturated fatty acids which helps to maintain membrane fluidity, probably within the lipid rafts domain, at the same time that a membrane resistance to oxidative damage, a trait observed in long-lived species, thus maintaining membrane functional integrity (7).

Interestingly, in yeast, sphingolipid biosynthesis inhibition reduces the activity of the sphingolipid-regulated Pkh1/2 protein kinases and one of their downstream targets, the Sch9 protein kinase resulting in an enhanced chronological life span (37). Pkh1 and 2 are functional homologs of the master kinase mammalian PDPK1 (phosphoinositide-dependent protein kinase 1), whereas Sch9 is related to mammalian S6K1 (ribosomal S6 protein kinase 1) (38), and both kinases are components of the nutrient-responsive mTORC1 (mammalian target of rapamycin complex 1) signaling pathway which play a key role in the aging process and determination of longevity (39–43). Although further studies are, however, needed to consolidate these new ideas in the human longevity context, it is hypothesized that the specific sphingolipidome profile shown by centenarians is a phenotypic trait that affect cell fluidity, organization, geometry, lipid–protein, and protein–protein interactions in a way that down-regulates signaling pathways such as mTOR which is ultimately associated with a longer life span.

In conclusion, our findings suggest the presence of a specific sphingolipidome in centenarians as phenotypic trait of this human population.

## Supplementary Material

glab360_suppl_Supplementary_Data
