# Supplementary material for: Long-lived Humans Have a Unique Plasma Sphingolipidome
Source: J Gerontol A Biol Sci Med Sci. 2021 Dec 5;77(4):728–35. doi: 10.1093/gerona/glab360 (PMC8974335; doi:10.1093/gerona/glab360)
Supplement: glab360_suppl_Supplementary_Data [file glab360_suppl_supplementary_data.pdf]

Supplemental data

Long-lived humans have a unique plasma sphingolipidome

Irene Pradas, PhD,[1,a] Mariona Jové, PhD,[1,a,c] Kevin Huynh, PhD,[2,a] Marta Ingles, PhD,[3] Consuelo Borras, PhD,[3] Natalia Mota-Martorell, PhD,[1] Jose Daniel Galo-Licona, MBMS,[1] Josep Puig, MD, PhD,[4] Jose Viña, MD, PhD,[3,b] Peter J. Meikle, PhD,[2,b] Reinald Pamplona, MD, PhD[1,b,*]

[1]Department of Experimental Medicine, University of Lleida-Lleida Biomedical Research Institute (UdL-IRBLleida), Lleida 25198, Catalonia, Spain.

[2]Baker Heart and Diabetes Institute, Melbourne VIC 3004, Australia.

[3]Department of Physiology, University of Valencia, Valencia 46004, Spain.

[4]Girona Biomedical Research Institute (IDIBGI), Hospital Universitari Dr Josep Trueta, Girona 17007, Catalonia, Spain.

[a] These authors contributed equally to this work

[b] Senior co-authors

[c] Serra Húnter Fellow

Supplementary Table 1. Conditions for tandem mass spectrometry quantification of major lipid species internal standards in targeted lipidomic analysis.

**Internal standard**	**Lipid Class**	**number of species**	**pmol***	**Transition**	**RT**	**TS**	**CE**
Sph(d17:1)	Sphingosine<br>Sphingosine-1-phosphate	3<br>5	1000	286.3 -> 268.3	2.06	3	8
SM(30:1)	Sphingomyelin	44	200	647.5 -> 184.1	4.50	2	25
dhCer(d18:0/8:0)	Dihydroceramide	5	100	428.4 -> 284.3	4.65	1	30
Cer(d18:1/17:0)	Ceramide<br>Ceramide-1-phosphate	55<br>1	100	552.5 -> 264.3	8.43	1	29
GlcCer(d18:1/16:0) *(d3)*	Monohexosylceramide	14	50	703.6 -> 264.3	6.36	1	33
LacCer(d18:1/16:0) *(d3)*	Dihexosylceramide	10	50	865.6 -> 264.3	5.84	1	53
Hex3Cer(d18:1/17:0)	Trihexosylceramide<br>Ganglioside	6<br>8	50	1038.7 -> 264.3	5.99	1	57
Sulfatide(d18:1/12:0)	Sulfatide	6	50	724.8 -> 264.3	4.10	3	56

*Amount of internal standard per sample; RT: retention time (minutes); TS: time segment (arb. unit); CE: collision energy (volts).

Supplementary Table 2. Sphingolipid species concentrations in plasma samples from adults, aged and centenarians.

**Lipid Species**	**Adults**	**Aged**	**Centenarians**
Sphingosine			
Sph(d16:1)	0.39 ± 0.08	0.36 ± 0.09	0.39 ± 0.09
Sph(d18:1)	1.1 ± 0.28	0.96 ± 0.22	1.07 ± 0.25
Sph(d18:2)	0.4 ± 0.09	0.34 ± 0.09	0.41 ± 0.12
Sphingosine 1 phosphate			
S1P(d16:1)	0.1 ± 0.04	0.09 ± 0.03	0.09 ± 0.03
S1P(d17:1)	0.03 ± 0.01	0.02 ± 0.01	0.03 ± 0.01
S1P(d18:0)	0.05 ± 0.02	0.04 ± 0.02	0.04 ± 0.02
S1P(d18:1)	0.55 ± 0.22	0.46 ± 0.18	0.55 ± 0.2
S1P(d18:2)	0.22 ± 0.1	0.18 ± 0.06	0.18 ± 0.08
Ceramides			
Cer(d16:1/16:0)	0.02 ± 0.01	0.02 ± 0.01	0.02 ± 0.01
Cer(d16:1/18:0)	0.02 ± 0.01	0.03 ± 0.01	0.02 ± 0.01
Cer(d16:1/20:0)	0.03 ± 0.01	0.03 ± 0.01	0.03 ± 0.01
Cer(d16:1/22:0)	0.31 ± 0.19	0.33 ± 0.14	0.23 ± 0.13
Cer(d16:1/23:0)	0.18 ± 0.12	0.19 ± 0.08	0.14 ± 0.08

Cer(d16:1/24:0)[*d]	0.68 ± 0.35 [c**]	0.64 ± 0.23 [c*]	0.43 ± 0.23
Cer(d16:1/24:1)	0.17 ± 0.1	0.2 ± 0.09	0.22 ± 0.11
Cer(d17:1/16:0)	0.02 ± 0.01	0.02 ± 0.01	0.02 ± 0.01
Cer(d17:1/18:0)	0.01 ± 0.003	0.01 ± 0.003	0.01 ± 0.004
Cer(d17:1/20:0)	0.01 ± 0.003	0.01 ± 0.003	0.01 ± 0.003
Cer(d17:1/22:0)	0.12 ± 0.07	0.13 ± 0.06	0.11 ± 0.05
Cer(d17:1/23:0)	0.11 ± 0.07	0.13 ± 0.06	0.11 ± 0.06
Cer(d17:1/24:0)	0.44 ± 0.26	0.43 ± 0.18	0.34 ± 0.16
Cer(d17:1/24:1)	0.18 ± 0.1	0.22 ± 0.1	0.27 ± 0.14
Cer(d18:1/14:0)	0.01 ± 0.005	0.01 ± 0.004	0.01 ± 0.01
Cer(d18:1/16:0)	0.31 ± 0.09	0.36 ± 0.13	0.44 ± 0.22
Cer(d18:1/18:0)	0.1 ± 0.04	0.13 ± 0.05	0.15 ± 0.08
Cer(d18:1/18:0) (a)	0.31 ± 0.07	0.3 ± 0.07	0.3 ± 0.05
Cer(d18:1/19:0)	0.02 ± 0.003	0.02 ± 0.003	0.02 ± 0.003
Cer(d18:1/20:0)	0.31 ± 0.12	0.4 ± 0.15	0.45 ± 0.22
Cer(d18:1/20:0) (a)	0.02 ± 0.01	0.02 ± 0.01	0.02 ± 0.01
Cer(d18:1/21:0)*[i]	0.05 ± 0.02	0.07 ± 0.03 [a*]	0.09 ± 0.04 [a***]
Cer(d18:1/22:0)	3.07 ± 1.37	3.37 ± 1.33	2.93 ± 1.4
Cer(d18:1/22:0) (a)	0.04 ± 0.01	0.06 ± 0.03	0.05 ± 0.02

Cer(d18:1/23:0)	2.57 ± 1.23	3.04 ± 1.17	2.54 ± 1.33
Cer(d18:1/24:0)	10.17 ± 4.85	10.13 ± 4.14	8.05 ± 4.13
Cer(d18:1/24:0) (a)	0.07 ± 0.02	0.1 ± 0.04	0.08 ± 0.04
Cer(d18:1/24:1)*[i]	3.27 ± 1.52 [c***]	4.29 ± 1.8 [c***]	6.08 ± 3.04
Cer(d18:1/24:1) (a)	0.03 ± 0.01	0.06 ± 0.03	0.07 ± 0.04
Cer(d18:1/26:0)	0.16 ± 0.08	0.16 ± 0.08	0.15 ± 0.05
Cer(d18:2/14:0)	0.003 ± 0.002	0.003 ± 0.001	0.003 ± 0.002
Cer(d18:2/16:0)	0.05 ± 0.02	0.06 ± 0.02	0.06 ± 0.03
Cer(d18:2/17:0)	0.01 ± 0.001	0.01 ± 0.002	0.01 ± 0.001
Cer(d18:2/18:0)	0.02 ± 0.01	0.03 ± 0.01	0.02 ± 0.01
Cer(d18:2/20:0)	0.02 ± 0.01	0.03 ± 0.01	0.02 ± 0.01
Cer(d18:2/21:0)	0.01 ± 0.01	0.01 ± 0.01	0.01 ± 0.01
Cer(d18:2/22:0)	0.53 ± 0.36	0.57 ± 0.26	0.45 ± 0.29
Cer(d18:2/23:0)	0.46 ± 0.28	0.59 ± 0.23	0.44 ± 0.31
Cer(d18:2/24:0)	1.95 ± 1.25	1.95 ± 0.82	1.42 ± 0.9
Cer(d18:2/24:1)	0.45 ± 0.27	0.55 ± 0.25	0.67 ± 0.42
Cer(d18:2/26:0)	0.03 ± 0.02	0.03 ± 0.01	0.02 ± 0.01
Cer(d19:1/16:0)	0.003 ± 0.002	0.003 ± 0.001	0.003 ± 0.002
Cer(d19:1/18:0)	0.003 ± 0.002	0.003 ± 0.002	0.004 ± 0.003

Cer(d19:1/20:0)	0.01 ± 0.01	0.01 ± 0.01	0.01 ± 0.01
Cer(d19:1/22:0)	0.16 ± 0.11	0.15 ± 0.07	0.13 ± 0.08
Cer(d19:1/23:0)	0.13 ± 0.1	0.14 ± 0.07	0.11 ± 0.06
Cer(d19:1/24:0)	0.54 ± 0.34	0.49 ± 0.24	0.37 ± 0.22
Cer(d19:1/24:1)	0.24 ± 0.17	0.27 ± 0.15	0.34 ± 0.21
Cer(d19:1/26:0)	0.01 ± 0.01	0.01 ± 0.004	0.01 ± 0.003
Cer(d20:1/22:0)	0.09 ± 0.02	0.09 ± 0.02	0.08 ± 0.02
Cer(d20:1/23:0)	0.02 ± 0.01	0.03 ± 0.01 [c**]	0.02 ± 0.01
Cer(d20:1/24:0)	0.09 ± 0.04 [c*]	0.09 ± 0.04 [c*]	0.06 ± 0.02
Cer(d20:1/24:1)	0.04 ± 0.02	0.04 ± 0.02	0.04 ± 0.03
Cer(d20:1/26:0)	0.02 ± 0.01	0.02 ± 0.01	0.02 ± 0.005
Ceramide 1 phosphate			
Cer1P(d18:1/16:0)	0.01 ± 0.003	0.01 ± 0.004	0.01 ± 0.01
Dihydroceramides			
Cer(d18:0/16:0)	0.03 ± 0.01	0.02 ± 0.01	0.02 ± 0.01
Cer(d18:0/18:0)	0.01 ± 0.01	0.02 ± 0.01	0.01 ± 0.01
Cer(d18:0/20:0)	0.06 ± 0.02	0.06 ± 0.02	0.05 ± 0.02
Cer(d18:0/22:0)*[d]	0.29 ± 0.09 [c***]	0.26 ± 0.1 [c**]	0.17 ± 0.05
Cer(d18:0/24:0)*[d]	0.42 ± 0.14 [c***]	0.37 ± 0.14 [c**]	0.24 ± 0.08

Cer(d18:0/24:1)	0.22 ± 0.08	0.26 ± 0.13	0.25 ± 0.09
Sphingomyelins			
SM(d17:1/14:0)	0.17 ± 0.07	0.18 ± 0.07	0.19 ± 0.08
SM(d18:0/14:0)	0.24 ± 0.09	0.25 ± 0.09	0.25 ± 0.09
SM(d18:1/14:0)/SM(d16:1/16:0)	9.73 ± 3.89	9.99 ± 3.19	9.78 ± 2.92
SM(d18:2/14:0)	0.73 ± 0.27	0.88 ± 0.33	0.83 ± 0.38
SM(d17:1/16:0)	5.4 ± 2.2	5.75 ± 2.12	6.18 ± 1.78
SM(d18:0/16:0)	3.96 ± 1.15	4.43 ± 1.7	4.68 ± 1.72
SM(d18:1/16:0)	125.99 ± 37.83	132.16 ± 45.48	151.71 ± 52.11
SM(d18:2/16:0)	17.01 ± 5.82	20.1 ± 6.93	20 ± 8.31
SM(34:3)	0.1 ± 0.04	0.11 ± 0.05	0.09 ± 0.03
SM(d16:1/19:0)	0.52 ± 0.24	0.46 ± 0.19	0.51 ± 0.22
SM(d18:1/17:0)/SM(d17:1/18:0)	2.21 ± 0.83	2.45 ± 0.85	2.43 ± 0.75
SM(d18:2/17:0)	0.41 ± 0.16	0.48 ± 0.19	0.44 ± 0.17
SM(35:2) (b)	0.17 ± 0.06	0.18 ± 0.07	0.21 ± 0.07
SM(d18:1/18:0)/SM(d16:1/20:0)	24.12 ± 9.05	24.65 ± 8.16	23.68 ± 7.91
SM(d18:2/18:0)	10.83 ± 4.56	12.28 ± 4.56	10.7 ± 4.41
SM(d18:2/18:1)	0.78 ± 0.28	0.82 ± 0.34	0.73 ± 0.31
SM(37:1)*[d]	4.5 ± 1.93	2.83 ± 1.16 [a***]	2.21 ± 0.89 [a***]

SM(37:2)	0.42 ± 0.16	0.39 ± 0.15	0.33 ± 0.11
SM(d18:1/20:0)/SM(d16:1/22:0)	16.04 ± 5.17	15.61 ± 4.92	12.51 ± 4.09
SM(d18:2/20:0)	5.8 ± 2.01	6.03 ± 2.1	4.94 ± 1.92
SM(38:3) (a)	0.31 ± 0.12	0.31 ± 0.12	0.35 ± 0.14
SM(38:3) (b) *[d]	0.13 ± 0.06 [c**]	0.1 ± 0.04	0.08 ± 0.03
SM(d16:1/23:0)/SM(d17:1/22:0)	5.05 ± 2.01 [c*]	5.1 ± 1.64 [c*]	3.75 ± 1.22
SM(d18:0/22:0)	0.43 ± 0.14 [c**]	0.47 ± 0.21 [c***]	0.28 ± 0.13
SM(d18:1/22:0)/SM(d16:1/24:0)	33.35 ± 9.4 [c*]	33.59 ± 11.89 [c*]	25.15 ± 10.45
SM(d16:1/24:1)	5.85 ± 2.26	6.14 ± 2.52	6.75 ± 1.99
SM(d18:2/22:0)	17.22 ± 6.6 [c*]	17.07 ± 5.63 [c*]	12.8 ± 5.79
SM(40:3) (a)	1.36 ± 0.49	1.41 ± 0.53	1.83 ± 0.74
SM(40:3) (b)	0.53 ± 0.22	0.48 ± 0.19	0.51 ± 0.19
SM(41:0)	0.29 ± 0.09	0.31 ± 0.11	0.23 ± 0.08
SM(41:1) (a)	1.18 ± 0.52 [c*]	1.14 ± 0.51 [c*]	0.78 ± 0.32
SM(d18:1/23:0)/SM(d17:1/24:0)	11.22 ± 3.41	12.39 ± 4.27 [c*]	9.07 ± 3.66
SM(d17:1/24:1)	3.54 ± 1.4	3.98 ± 1.64	4.73 ± 1.34
SM(d18:2/23:0)	5.28 ± 2.01	5.6 ± 1.92 [c*]	4.06 ± 1.7
SM(d18:1/24:0)	22.27 ± 6.53	22.12 ± 8.57	16.84 ± 7.86
SM(d18:1/24:1)	39.21 ± 12.12 [c**]	46.24 ± 18.79	59.01 ± 24.35

SM(d18:2/24:0)	13.71 ± 5.17	13.18 ± 4.53	10.22 ± 4.61
SM(43:1)	1.13 ± 0.44	1.17 ± 0.54	0.9 ± 0.29
SM(d19:1/24:1)	1.07 ± 0.54	1.09 ± 0.54	1.24 ± 0.62
SM(43:2) (b)*[i]	0.45 ± 0.16 [c**]	0.53 ± 0.23	0.66 ± 0.19
SM(43:2) (c)	0.4 ± 0.15	0.44 ± 0.22	0.37 ± 0.13
SM(44:1)	0.22 ± 0.07	0.22 ± 0.09	0.19 ± 0.07
SM(44:2)	0.35 ± 0.11	0.39 ± 0.17	0.45 ± 0.17
SM(44:3)	0.3 ± 0.1 [c**]	0.32 ± 0.13 [c**]	0.47 ± 0.21
Monohexosylceramides			
Hex1Cer(d16:1/18:0)	0.1 ± 0.05	0.1 ± 0.05	0.11 ± 0.06
Hex1Cer(d16:1/20:0)	0.08 ± 0.03	0.08 ± 0.03	0.08 ± 0.04
Hex1Cer(d16:1/22:0)	0.37 ± 0.13	0.35 ± 0.15	0.35 ± 0.13
Hex1Cer(d16:1/24:0)	0.22 ± 0.08	0.22 ± 0.1	0.23 ± 0.1
Hex1Cer(d18:1/16:0)	4.86 ± 1.62 [c***]	4.76 ± 2.01 [c***]	7.58 ± 2.23
Hex1Cer(d18:1/18:0)	0.46 ± 0.18	0.45 ± 0.19	0.6 ± 0.23
Hex1Cer(d18:1/20:0)	0.28 ± 0.1	0.28 ± 0.13	0.36 ± 0.13
Hex1Cer(d18:1/22:0)	3.47 ± 1.16	3.17 ± 1.44	3.68 ± 1.41
Hex1Cer(d18:1/24:0)	5.74 ± 1.82	5.22 ± 2.16	6.45 ± 2.99
Hex1Cer(d18:1/24:1)	3.69 ± 1.38 [c***]	3.68 ± 1.62 [c***]	6.84 ± 2.72

Hex1Cer(d18:2/18:0)	0.08 ± 0.04	0.08 ± 0.03	0.08 ± 0.03
Hex1Cer(d18:2/20:0)	0.07 ± 0.03	0.07 ± 0.03	0.07 ± 0.04
Hex1Cer(d18:2/22:0)	0.45 ± 0.2	0.42 ± 0.18	0.46 ± 0.19
Hex1Cer(d18:2/24:0)	0.94 ± 0.44	0.87 ± 0.35	1.03 ± 0.46
Dihexosylceramides			
Hex2Cer(d16:1/16:0)	0.64 ± 0.2	0.64 ± 0.27	0.75 ± 0.24
Hex2Cer(d16:1/24:1)	0.07 ± 0.03	0.07 ± 0.04	0.08 ± 0.04
Hex2Cer(d18:1/16:0)	7.91 ± 2.56	7.25 ± 2.78	9.07 ± 3.52
Hex2Cer(d18:1/18:0)	0.1 ± 0.03	0.09 ± 0.05	0.11 ± 0.06
Hex2Cer(d18:1/20:0)	0.07 ± 0.03	0.08 ± 0.04	0.08 ± 0.04
Hex2Cer(d18:1/22:0)	0.45 ± 0.18	0.49 ± 0.2	0.48 ± 0.21
Hex2Cer(d18:1/24:0)	0.76 ± 0.26	0.78 ± 0.25	0.77 ± 0.31
Hex2Cer(d18:1/24:1)	1.53 ± 0.6	1.63 ± 0.74	2.18 ± 1.1
Hex2Cer(d18:2/16:0)	0.52 ± 0.19	0.53 ± 0.23	0.65 ± 0.26
Hex2Cer(d18:2/24:1)	0.37 ± 0.16	0.4 ± 0.22	0.5 ± 0.25
Trihexosylceramides			
Hex3Cer(d18:1/16:0)	1.25 ± 0.43 [c**]	1.28 ± 0.5 [c**]	1.82 ± 0.66
Hex3Cer(d18:1/18:0)	0.25 ± 0.1 [c**]	0.24 ± 0.13 [c**]	0.36 ± 0.13
Hex3Cer(d18:1/20:0)	0.12 ± 0.05 [c**]	0.12 ± 0.05 [c**]	0.17 ± 0.07

Hex3Cer(d18:1/22:0)	0.23 ± 0.08	0.24 ± 0.11	0.3 ± 0.14
Hex3Cer(d18:1/24:0)	0.19 ± 0.08	0.19 ± 0.08	0.24 ± 0.11
Hex3Cer(d18:1/24:1)	0.38 ± 0.14 [c***]	0.44 ± 0.22 [c***]	0.78 ± 0.3
Gangliosides			
GM1(d18:1/16:0)	0.05 ± 0.01 [c***]	0.04 ± 0.02 [c**]	0.06 ± 0.02
GM3(d18:1/16:0)	0.97 ± 0.33	1.03 ± 0.4	1.47 ± 0.53
GM3(d18:1/18:0)	0.37 ± 0.14	0.35 ± 0.13	0.32 ± 0.12
GM3(d18:1/20:0)	0.13 ± 0.06	0.15 ± 0.07	0.19 ± 0.07
GM3(d18:1/22:0)	0.68 ± 0.3	0.77 ± 0.31	0.81 ± 0.38
GM3(d18:1/24:0)	0.34 ± 0.15	0.32 ± 0.12	0.36 ± 0.17
GM3(d18:1/24:1)*[i]	0.7 ± 0.31 [c***]	0.83 ± 0.37 [c***]	1.33 ± 0.56
GM3(d18:2/24:1)*[i]	0.25 ± 0.11 [c***]	0.28 ± 0.13 [c**]	0.42 ± 0.15
Sulfatides			
Sulfatide (d18:1:/16:0(OH))	0.15 ± 0.07	0.12 ± 0.06	0.1 ± 0.07
Sulfatide (d18:1:/16:0)	0.13 ± 0.06	0.12 ± 0.06	0.1 ± 0.07
Sulfatide (d18:1:/24:0(OH))	0.07 ± 0.04 [c*]	0.07 ± 0.04 [c*]	0.04 ± 0.03
Sulfatide (d18:1:/24:0)	0.04 ± 0.02	0.03 ± 0.01	0.03 ± 0.02
Sulfatide (d18:1:/24:1(OH))	0.07 ± 0.03	0.06 ± 0.03	0.05 ± 0.04
Sulfatide (d18:1:/24:1)	0.06 ± 0.03	0.05 ± 0.03	0.06 ± 0.05

Sphingolipids (151 species); Sphingosine (3species); Sphingosine 1 phosphate (4species); Ceramides (49 species); Ceramide 1 phosphate (1 species); Dihydroceramides (6 species); Sphingomyelins (44 species); Monohexosylceramides (14 species); Dihexosylceramides (10 species); Trihexosylceramides (6 species); Gangliosides (8 species); Sulfatides (6 species).

Statistical analysis: One-way Anova post hoc Tukey's.

Concentrations in nmol/ml; Values: mean ± SD

Species separated chromatographically but incompletely characterized were labelled with (a) and (b), where (a) and (b) represent the elution order.

[a] Respect to Adults, [b] Respect to Aged, [c] Respect to Centenarians.

*d: decrease with age; *i: increase with age.

* $p< 0.05$; ** $p<0.01$; ***$p<0.001$

Supplementary Table 3. Multinomial linear regression model examining the association between sphingolipid species and the experimental groups (adults, aged and centenarians).

Lipids	Coefficient	Lower CI (95%)	Upper CI (95%)	p-value	p-value (BH)
**Adults vs Aged**					
Cer(d18:0/16:0)	0.8491	0.6154	1.1715	3.09E-01	9.45E-01
Cer(d18:0/18:0)	1.3587	0.9610	1.9212	8.11E-02	9.45E-01
Cer(d18:0/20:0)	0.9995	0.7808	1.2794	9.97E-01	1.00E+00
Cer(d18:0/22:0)	0.8582	0.6580	1.1193	2.51E-01	9.45E-01
Cer(d18:0/24:0)	0.8394	0.6449	1.0926	1.87E-01	9.45E-01
Cer(d18:0/24:1)	1.0678	0.7809	1.4602	6.73E-01	9.45E-01
Cer(d16:1/16:0)	1.1141	0.8137	1.5254	4.90E-01	9.45E-01
Cer(d16:1/18:0)	1.5054	1.0600	2.1381	2.35E-02	9.45E-01
Cer(d16:1/20:0)	1.2064	0.8847	1.6450	2.28E-01	9.45E-01
Cer(d16:1/22:0)	1.1060	0.7856	1.5570	5.54E-01	9.45E-01
Cer(d16:1/23:0)	1.1884	0.8413	1.6787	3.18E-01	9.45E-01
Cer(d16:1/24:0)	0.9666	0.7089	1.3180	8.25E-01	9.45E-01
Cer(d16:1/24:1)	1.3165	0.9214	1.8810	1.27E-01	9.45E-01
Cer(d17:1/16:0)	1.1374	0.8520	1.5184	3.72E-01	9.45E-01
Cer(d17:1/18:0)	1.2063	0.9372	1.5527	1.41E-01	9.45E-01
Cer(d17:1/20:0)	1.4427	1.0208	2.0390	3.85E-02	9.45E-01
Cer(d17:1/22:0)	1.1360	0.8106	1.5920	4.48E-01	9.45E-01
Cer(d17:1/23:0)	1.2594	0.8936	1.7751	1.81E-01	9.45E-01
Cer(d17:1/24:0)	0.9971	0.7058	1.4086	9.87E-01	1.00E+00
Cer(d17:1/24:1)	1.3234	0.9254	1.8927	1.21E-01	9.45E-01
Cer(d18:1/14:0)	1.1150	0.7667	1.6215	5.59E-01	9.45E-01
Cer(d18:1/16:0)	1.0656	0.8242	1.3777	6.19E-01	9.45E-01
Cer(d18:1/18:0)	1.3726	1.0091	1.8672	4.40E-02	9.45E-01
Cer(d18:1/19:0)	1.0535	0.9449	1.1746	3.38E-01	9.45E-01
Cer(d18:1/20:0)	1.2893	0.9654	1.7219	8.34E-02	9.45E-01
Cer(d18:1/21:0)	1.4750	1.0834	2.0080	1.50E-02	9.45E-01
Cer(d18:1/22:0)	1.0420	0.7775	1.3967	7.77E-01	9.45E-01
Cer(d18:1/23:0)	1.1099	0.8276	1.4885	4.76E-01	9.45E-01
Cer(d18:1/24:0)	0.9382	0.7001	1.2573	6.61E-01	9.45E-01
Cer(d18:1/24:1)	1.2675	0.9115	1.7625	1.53E-01	9.45E-01
Cer(d18:1/26:0)	0.9266	0.6650	1.2911	6.44E-01	9.45E-01
Cer(d18:2/14:0)	1.2378	0.7491	2.0453	3.95E-01	9.45E-01
Cer(d18:2/16:0)	1.1841	0.8844	1.5854	2.48E-01	9.45E-01
Cer(d18:2/17:0)	1.0412	0.8610	1.2591	6.69E-01	9.45E-01
Cer(d18:2/18:0)	1.4122	0.9760	2.0434	6.62E-02	9.45E-01
Cer(d18:2/20:0)	1.2125	0.8506	1.7284	2.78E-01	9.45E-01

Cer(d18:2/21:0)	1.4464	0.9817	2.1312	6.13E-02	9.45E-01
Cer(d18:2/22:0)	1.1249	0.7717	1.6396	5.30E-01	9.45E-01
Cer(d18:2/23:0)	1.2959	0.9182	1.8291	1.36E-01	9.45E-01
Cer(d18:2/24:0)	1.0000	0.7077	1.4132	1.00E+00	1.00E+00
Cer(d18:2/24:1)	1.3059	0.8920	1.9119	1.64E-01	9.45E-01
Cer(d18:2/26:0)	0.9180	0.6404	1.3159	6.33E-01	9.45E-01
Cer(d19:1/16:0)	0.9847	0.6436	1.5065	9.42E-01	9.81E-01
Cer(d19:1/18:0)	1.2757	0.7632	2.1325	3.43E-01	9.45E-01
Cer(d19:1/20:0)	1.1550	0.7709	1.7305	4.75E-01	9.45E-01
Cer(d19:1/22:0)	0.9993	0.6723	1.4854	9.97E-01	1.00E+00
Cer(d19:1/23:0)	1.1164	0.7299	1.7074	6.02E-01	9.45E-01
Cer(d19:1/24:0)	0.9270	0.6096	1.4096	7.16E-01	9.45E-01
Cer(d19:1/24:1)	1.2014	0.7513	1.9211	4.33E-01	9.45E-01
Cer(d19:1/26:0)	0.8800	0.6309	1.2272	4.41E-01	9.45E-01
Cer(d20:1/22:0)	0.9751	0.7942	1.1972	8.04E-01	9.45E-01
Cer(d20:1/23:0)	1.1933	0.8519	1.6717	2.95E-01	9.45E-01
Cer(d20:1/24:0)	0.9211	0.6602	1.2850	6.20E-01	9.45E-01
Cer(d20:1/24:1)	1.1439	0.7496	1.7458	5.23E-01	9.45E-01
Cer(d20:1/26:0)	0.8710	0.6415	1.1826	3.66E-01	9.45E-01
Cer1P(d18:1/16:0)	0.9872	0.7581	1.2855	9.22E-01	9.66E-01
GM1(d18:1/16:0)	1.0410	0.7851	1.3802	7.74E-01	9.45E-01
GM3(d18:1/16:0)	1.0715	0.8206	1.3991	6.03E-01	9.45E-01
GM3(d18:1/18:0)	0.9575	0.7208	1.2718	7.58E-01	9.45E-01
GM3(d18:1/20:0)	1.0968	0.7719	1.5585	5.97E-01	9.45E-01
GM3(d18:1/22:0)	1.1108	0.8234	1.4986	4.81E-01	9.45E-01
GM3(d18:1/24:0)	0.9670	0.7135	1.3105	8.24E-01	9.45E-01
GM3(d18:1/24:1)	1.1531	0.8354	1.5917	3.76E-01	9.45E-01
GM3(d18:2/24:1)	1.2197	0.8900	1.6715	2.09E-01	9.45E-01
Hex1Cer(d16:1/18:0)	0.9724	0.6840	1.3825	8.73E-01	9.61E-01
Hex1Cer(d16:1/20:0)	1.1039	0.7913	1.5400	5.51E-01	9.45E-01
Hex1Cer(d16:1/22:0)	0.9843	0.7455	1.2996	9.09E-01	9.65E-01
Hex1Cer(d16:1/24:0)	1.0317	0.7726	1.3776	8.28E-01	9.45E-01
Hex1Cer(d18:1/16:0)	0.9052	0.6908	1.1861	4.60E-01	9.45E-01
Hex1Cer(d18:1/18:0)	0.9369	0.7138	1.2295	6.30E-01	9.45E-01
Hex1Cer(d18:1/20:0)	0.9132	0.6906	1.2074	5.14E-01	9.45E-01
Hex1Cer(d18:1/22:0)	0.8265	0.6126	1.1153	2.05E-01	9.45E-01
Hex1Cer(d18:1/24:0)	0.8256	0.6352	1.0730	1.47E-01	9.45E-01
Hex1Cer(d18:1/24:1)	0.9441	0.7132	1.2497	6.80E-01	9.45E-01
Hex1Cer(d18:2/18:0)	1.0065	0.7558	1.3402	9.64E-01	9.97E-01
Hex1Cer(d18:2/20:0)	1.1380	0.8523	1.5195	3.71E-01	9.45E-01
Hex1Cer(d18:2/22:0)	0.9224	0.6886	1.2356	5.79E-01	9.45E-01
Hex1Cer(d18:2/24:0)	0.9347	0.7056	1.2382	6.29E-01	9.45E-01
Hex2Cer(d16:1/16:0)	1.0187	0.7775	1.3347	8.90E-01	9.65E-01
Hex2Cer(d16:1/24:1)	1.0445	0.7197	1.5159	8.14E-01	9.45E-01
Hex2Cer(d18:1/16:0)	0.9150	0.7019	1.1927	5.01E-01	9.45E-01

Hex2Cer(d18:1/18:0)	0.8304	0.6186	1.1148	2.09E-01	9.45E-01
Hex2Cer(d18:1/20:0)	1.1604	0.8964	1.5020	2.50E-01	9.45E-01
Hex2Cer(d18:1/22:0)	1.1613	0.8959	1.5053	2.50E-01	9.45E-01
Hex2Cer(d18:1/24:0)	1.0891	0.8535	1.3898	4.82E-01	9.45E-01
Hex2Cer(d18:1/24:1)	1.0446	0.7730	1.4117	7.70E-01	9.45E-01
Hex2Cer(d18:2/16:0)	1.0044	0.7579	1.3311	9.75E-01	1.00E+00
Hex2Cer(d18:2/24:1)	1.0637	0.7695	1.4703	7.01E-01	9.45E-01
Hex3Cer(d18:1/16:0)	1.0299	0.7865	1.3488	8.26E-01	9.45E-01
Hex3Cer(d18:1/18:0)	1.0309	0.7101	1.4967	8.69E-01	9.61E-01
Hex3Cer(d18:1/20:0)	1.0639	0.8020	1.4112	6.59E-01	9.45E-01
Hex3Cer(d18:1/22:0)	1.0445	0.7934	1.3750	7.50E-01	9.45E-01
Hex3Cer(d18:1/24:0)	1.0202	0.7636	1.3630	8.90E-01	9.65E-01
Hex3Cer(d18:1/24:1)	1.1301	0.8426	1.5157	4.04E-01	9.45E-01
S1P(d16:1)	0.9140	0.6711	1.2447	5.58E-01	9.45E-01
S1P(d17:1)	0.9423	0.6717	1.3219	7.24E-01	9.45E-01
S1P(d18:0)	0.8824	0.6457	1.2060	4.22E-01	9.45E-01
S1P(d18:1)	0.8425	0.6271	1.1320	2.47E-01	9.45E-01
S1P(d18:2)	0.8777	0.6535	1.1787	3.76E-01	9.45E-01
Sph(d16:1)	0.9035	0.7546	1.0818	2.61E-01	9.45E-01
Sph(d18:1)	0.8721	0.7254	1.0485	1.41E-01	9.45E-01
Sph(d18:2)	0.8435	0.6964	1.0217	8.01E-02	9.45E-01
SM(d17:1/14:0)	1.1260	0.8589	1.4762	3.80E-01	9.45E-01
SM(d18:0/14:0)	1.0401	0.7978	1.3560	7.65E-01	9.45E-01
SM(d18:1/14:0)/ SM(d16:1/16:0)	1.0932	0.8638	1.3834	4.48E-01	9.45E-01
SM(d18:2/14:0)	1.2220	0.9534	1.5662	1.10E-01	9.45E-01
SM(d17:1/16:0)	1.1213	0.8547	1.4711	3.98E-01	9.45E-01
SM(d18:0/16:0)	1.0984	0.8608	1.4016	4.40E-01	9.45E-01
SM(d18:1/16:0)	1.0244	0.8135	1.2900	8.33E-01	9.45E-01
SM(d18:2/16:0)	1.1952	0.9323	1.5324	1.54E-01	9.45E-01
SM(34:3)	1.1208	0.8380	1.4991	4.32E-01	9.45E-01
SM(d16:1/19:0)	0.9440	0.6764	1.3175	7.28E-01	9.45E-01
SM(d18:1/17:0)/ SM(d17:1/18:0)	1.1344	0.8711	1.4773	3.39E-01	9.45E-01
SM(d18:2/17:0)	1.2027	0.9032	1.6014	1.99E-01	9.45E-01
SM(35:2) (b)	1.0912	0.8306	1.4335	5.21E-01	9.45E-01
SM(d18:1/18:0)/ SM(d16:1/20:0)	1.0314	0.7971	1.3347	8.09E-01	9.45E-01
SM(d18:2/18:0)	1.1606	0.8749	1.5397	2.92E-01	9.45E-01
SM(d18:2/18:1)	1.0317	0.7716	1.3793	8.29E-01	9.45E-01
SM(37:1)	0.6684	0.4969	0.8993	9.18E-03	9.45E-01
SM(37:2)	0.9843	0.7650	1.2663	8.99E-01	9.65E-01
SM(d18:1/20:0)/ SM(d16:1/22:0)	0.9805	0.7772	1.2369	8.64E-01	9.61E-01
SM(d18:2/20:0)	1.0574	0.8185	1.3659	6.61E-01	9.45E-01
SM(38:3) (a)	1.0229	0.7724	1.3547	8.71E-01	9.61E-01
SM(38:3) (b)	0.8840	0.6641	1.1767	3.88E-01	9.45E-01

SM(d16:1/23:0)/ SM(d17:1/22:0)	1.0497	0.8227	1.3393	6.89E-01	9.45E-01
SM(d18:0/22:0)	1.0493	0.7752	1.4203	7.49E-01	9.45E-01
SM(d18:1/22:0)/ SM(d16:1/24:0)	0.9508	0.7543	1.1985	6.61E-01	9.45E-01
SM(d16:1/24:1)	1.0823	0.8038	1.4572	5.93E-01	9.45E-01
SM(d18:2/22:0)	1.0155	0.7897	1.3059	9.02E-01	9.65E-01
SM(40:3) (a)	1.0413	0.7932	1.3670	7.65E-01	9.45E-01
SM(40:3) (b)	0.9601	0.7247	1.2718	7.70E-01	9.45E-01
SM(41:0)	1.0498	0.8150	1.3523	6.99E-01	9.45E-01
SM(41:1) (a)	0.9601	0.6881	1.3397	8.06E-01	9.45E-01
SM(d18:1/23:0)/ SM(d17:1/24:0)	1.0471	0.8319	1.3179	6.88E-01	9.45E-01
SM(d17:1/24:1)	1.1544	0.8625	1.5451	3.24E-01	9.45E-01
SM(d18:2/23:0)	1.0820	0.8471	1.3819	5.18E-01	9.45E-01
SM(d18:1/24:0)	0.9199	0.7213	1.1733	4.91E-01	9.45E-01
SM(d18:1/24:1)	1.1259	0.8472	1.4963	4.03E-01	9.45E-01
SM(d18:2/24:0)	0.9694	0.7534	1.2473	8.04E-01	9.45E-01
SM(43:1)	0.9831	0.7137	1.3542	9.15E-01	9.65E-01
SM(d19:1/24:1)	1.0566	0.7035	1.5868	7.85E-01	9.45E-01
SM(43:2) (a)	1.1163	0.8317	1.4982	4.53E-01	9.45E-01
SM(43:2) (b)	1.0715	0.7897	1.4538	6.49E-01	9.45E-01
SM(44:1)	0.9341	0.7012	1.2444	6.33E-01	9.45E-01
SM(44:2)	1.0483	0.7802	1.4086	7.48E-01	9.45E-01
SM(44:3)	1.0312	0.7779	1.3672	8.26E-01	9.45E-01
Sulfatide (d18:1:/16:0(OH))	0.8213	0.5986	1.1271	2.15E-01	9.45E-01
Sulfatide (d18:1:/16:0)	0.9125	0.6871	1.2117	5.17E-01	9.45E-01
Sulfatide (d18:1:/24:0(OH))	0.9616	0.6752	1.3696	8.24E-01	9.45E-01
Sulfatide (d18:1:/24:0)	0.8858	0.6486	1.2099	4.35E-01	9.45E-01
Sulfatide (d18:1:/24:1(OH))	0.7756	0.5574	1.0792	1.27E-01	9.45E-01
Sulfatide (d18:1:/24:1)	0.8955	0.6441	1.2451	5.01E-01	9.45E-01
**Adults vs Centenarians**					
Cer(d18:0/16:0)	1.0142	0.7645	1.3454	9.20E-01	9.67E-01
Cer(d18:0/18:0)	0.7834	0.5120	1.1988	2.53E-01	4.81E-01
Cer(d18:0/20:0)	0.9265	0.7226	1.1881	5.39E-01	7.06E-01
Cer(d18:0/22:0)	0.7586	0.5991	0.9607	2.30E-02	1.66E-01
Cer(d18:0/24:0)	0.7639	0.5948	0.9812	3.56E-02	1.93E-01
Cer(d18:0/24:1)	1.0909	0.8089	1.4713	5.60E-01	7.21E-01
Cer(d16:1/16:0)	1.0321	0.7735	1.3770	8.26E-01	9.16E-01
Cer(d16:1/18:0)	0.8151	0.5762	1.1531	2.41E-01	4.64E-01
Cer(d16:1/20:0)	0.8981	0.6671	1.2092	4.70E-01	6.80E-01
Cer(d16:1/22:0)	0.7710	0.5566	1.0679	1.15E-01	3.55E-01
Cer(d16:1/23:0)	0.7906	0.5826	1.0728	1.28E-01	3.56E-01
Cer(d16:1/24:0)	0.7379	0.5459	0.9975	4.82E-02	2.19E-01
Cer(d16:1/24:1)	1.1637	0.8266	1.6382	3.76E-01	6.20E-01
Cer(d17:1/16:0)	1.1131	0.8378	1.4789	4.51E-01	6.59E-01

Cer(d17:1/18:0)	0.9785	0.7639	1.2533	8.60E-01	9.27E-01
Cer(d17:1/20:0)	0.9923	0.7471	1.3178	9.56E-01	9.82E-01
Cer(d17:1/22:0)	0.9257	0.6767	1.2663	6.21E-01	7.68E-01
Cer(d17:1/23:0)	0.8733	0.6327	1.2054	4.01E-01	6.26E-01
Cer(d17:1/24:0)	0.8746	0.6359	1.2030	4.01E-01	6.26E-01
Cer(d17:1/24:1)	1.3109	0.9281	1.8516	1.21E-01	3.55E-01
Cer(d18:1/14:0)	1.2759	0.8920	1.8252	1.77E-01	3.99E-01
Cer(d18:1/16:0)	1.3444	1.0081	1.7928	4.42E-02	2.19E-01
Cer(d18:1/18:0)	1.1781	0.8388	1.6548	3.36E-01	5.75E-01
Cer(d18:1/19:0)	1.0324	0.9425	1.1310	4.83E-01	6.86E-01
Cer(d18:1/20:0)	1.2128	0.8945	1.6443	2.08E-01	4.33E-01
Cer(d18:1/21:0)	1.3082	0.9702	1.7641	7.69E-02	2.66E-01
Cer(d18:1/22:0)	0.9997	0.7594	1.3160	9.98E-01	9.98E-01
Cer(d18:1/23:0)	0.9662	0.7267	1.2846	8.08E-01	9.16E-01
Cer(d18:1/24:0)	0.9247	0.6947	1.2309	5.83E-01	7.33E-01
Cer(d18:1/24:1)	1.5546	1.1197	2.1583	9.64E-03	1.13E-01
Cer(d18:1/26:0)	1.1354	0.8650	1.4905	3.51E-01	5.93E-01
Cer(d18:2/14:0)	0.9511	0.5916	1.5290	8.32E-01	9.16E-01
Cer(d18:2/16:0)	1.0698	0.7994	1.4318	6.42E-01	7.88E-01
Cer(d18:2/17:0)	1.1225	0.9466	1.3312	1.78E-01	3.99E-01
Cer(d18:2/18:0)	0.9734	0.6535	1.4498	8.92E-01	9.50E-01
Cer(d18:2/20:0)	0.9676	0.6862	1.3644	8.48E-01	9.26E-01
Cer(d18:2/21:0)	1.1367	0.8093	1.5966	4.51E-01	6.59E-01
Cer(d18:2/22:0)	0.8554	0.5960	1.2277	3.88E-01	6.21E-01
Cer(d18:2/23:0)	0.8097	0.5685	1.1533	2.35E-01	4.64E-01
Cer(d18:2/24:0)	0.8048	0.5699	1.1365	2.11E-01	4.33E-01
Cer(d18:2/24:1)	1.3169	0.9056	1.9150	1.45E-01	3.74E-01
Cer(d18:2/26:0)	1.0380	0.7540	1.4290	8.15E-01	9.16E-01
Cer(d19:1/16:0)	1.2197	0.7472	1.9909	4.18E-01	6.26E-01
Cer(d19:1/18:0)	1.1560	0.6877	1.9432	5.76E-01	7.30E-01
Cer(d19:1/20:0)	1.1463	0.7601	1.7286	5.06E-01	6.87E-01
Cer(d19:1/22:0)	0.8820	0.5674	1.3710	5.69E-01	7.26E-01
Cer(d19:1/23:0)	0.8356	0.5373	1.2995	4.16E-01	6.26E-01
Cer(d19:1/24:0)	0.8019	0.5079	1.2659	3.34E-01	5.75E-01
Cer(d19:1/24:1)	1.2694	0.7733	2.0836	3.37E-01	5.75E-01
Cer(d19:1/26:0)	1.0101	0.7694	1.3260	9.41E-01	9.76E-01
Cer(d20:1/22:0)	0.9391	0.7697	1.1458	5.27E-01	6.97E-01
Cer(d20:1/23:0)	0.7332	0.5235	1.0271	7.02E-02	2.61E-01
Cer(d20:1/24:0)	0.8021	0.5875	1.0950	1.60E-01	3.99E-01
Cer(d20:1/24:1)	1.1853	0.8053	1.7448	3.80E-01	6.20E-01
Cer(d20:1/26:0)	0.9146	0.7345	1.1389	4.16E-01	6.26E-01
Cer1P(d18:1/16:0)	1.3976	1.0420	1.8746	2.65E-02	1.68E-01
GM1(d18:1/16:0)	1.3740	1.0487	1.8004	2.23E-02	1.66E-01
GM3(d18:1/16:0)	1.5934	1.2205	2.0802	1.05E-03	3.18E-02
GM3(d18:1/18:0)	1.0315	0.7721	1.3782	8.30E-01	9.16E-01

GM3(d18:1/20:0)	1.5122	1.1256	2.0318	7.20E-03	9.36E-02
GM3(d18:1/22:0)	1.2292	0.8855	1.7062	2.11E-01	4.33E-01
GM3(d18:1/24:0)	1.2503	0.9048	1.7278	1.71E-01	3.99E-01
GM3(d18:1/24:1)	1.8651	1.3664	2.5458	2.25E-04	8.54E-03
GM3(d18:2/24:1)	1.4814	1.0992	1.9965	1.11E-02	1.21E-01
Hex1Cer(d16:1/18:0)	1.1178	0.7972	1.5673	5.10E-01	6.87E-01
Hex1Cer(d16:1/20:0)	1.0826	0.7903	1.4830	6.13E-01	7.64E-01
Hex1Cer(d16:1/22:0)	1.0929	0.8275	1.4434	5.22E-01	6.97E-01
Hex1Cer(d16:1/24:0)	1.0513	0.7731	1.4295	7.44E-01	8.63E-01
Hex1Cer(d18:1/16:0)	1.7931	1.3914	2.3106	3.43E-05	1.74E-03
Hex1Cer(d18:1/18:0)	1.5225	1.1360	2.0404	5.99E-03	9.11E-02
Hex1Cer(d18:1/20:0)	1.5263	1.1627	2.0036	3.14E-03	5.30E-02
Hex1Cer(d18:1/22:0)	1.3727	1.0352	1.8201	2.87E-02	1.70E-01
Hex1Cer(d18:1/24:0)	1.3831	1.0443	1.8317	2.47E-02	1.66E-01
Hex1Cer(d18:1/24:1)	2.0712	1.5471	2.7727	9.84E-06	1.49E-03
Hex1Cer(d18:2/18:0)	1.3020	1.0038	1.6889	4.69E-02	2.19E-01
Hex1Cer(d18:2/20:0)	1.1719	0.8725	1.5740	2.84E-01	5.20E-01
Hex1Cer(d18:2/22:0)	1.2415	0.9465	1.6283	1.15E-01	3.55E-01
Hex1Cer(d18:2/24:0)	1.2834	0.9773	1.6852	7.16E-02	2.61E-01
Hex2Cer(d16:1/16:0)	1.2306	0.9371	1.6161	1.32E-01	3.56E-01
Hex2Cer(d16:1/24:1)	1.3314	0.9238	1.9189	1.21E-01	3.55E-01
Hex2Cer(d18:1/16:0)	1.3459	1.0086	1.7958	4.38E-02	2.19E-01
Hex2Cer(d18:1/18:0)	1.4196	1.0043	2.0066	4.73E-02	2.19E-01
Hex2Cer(d18:1/20:0)	0.9893	0.7266	1.3471	9.44E-01	9.76E-01
Hex2Cer(d18:1/22:0)	0.9943	0.7300	1.3542	9.70E-01	9.90E-01
Hex2Cer(d18:1/24:0)	1.0021	0.7607	1.3202	9.88E-01	9.94E-01
Hex2Cer(d18:1/24:1)	1.4412	1.0400	1.9971	2.90E-02	1.70E-01
Hex2Cer(d18:2/16:0)	1.3266	0.9713	1.8118	7.44E-02	2.63E-01
Hex2Cer(d18:2/24:1)	1.4045	0.9971	1.9782	5.19E-02	2.19E-01
Hex3Cer(d18:1/16:0)	1.5727	1.1978	2.0649	1.70E-03	4.31E-02
Hex3Cer(d18:1/18:0)	1.5479	1.0878	2.2026	1.64E-02	1.55E-01
Hex3Cer(d18:1/20:0)	1.5861	1.1806	2.1308	3.00E-03	5.30E-02
Hex3Cer(d18:1/22:0)	1.3434	0.9980	1.8082	5.15E-02	2.19E-01
Hex3Cer(d18:1/24:0)	1.3954	1.0448	1.8636	2.51E-02	1.66E-01
Hex3Cer(d18:1/24:1)	1.9926	1.4797	2.6834	3.14E-05	1.74E-03
S1P(d16:1)	1.1148	0.8514	1.4598	4.20E-01	6.26E-01
S1P(d17:1)	1.4162	1.0348	1.9381	3.06E-02	1.72E-01
S1P(d18:0)	1.2545	0.8998	1.7491	1.76E-01	3.99E-01
S1P(d18:1)	1.3768	1.0612	1.7863	1.73E-02	1.55E-01
S1P(d18:2)	1.1731	0.8857	1.5537	2.58E-01	4.84E-01
Sph(d16:1)	1.1104	0.9310	1.3245	2.37E-01	4.64E-01
Sph(d18:1)	1.1265	0.9478	1.3389	1.71E-01	3.99E-01
Sph(d18:2)	1.1654	0.9563	1.4204	1.26E-01	3.56E-01
SM(d17:1/14:0)	1.0874	0.8184	1.4448	5.55E-01	7.21E-01
SM(d18:0/14:0)	1.1386	0.8799	1.4735	3.15E-01	5.63E-01

SM(d18:1/14:0)/ SM(d16:1/16:0)	1.0506	0.8363	1.3198	6.64E-01	7.91E-01
SM(d18:2/14:0)	1.0283	0.7912	1.3365	8.31E-01	9.16E-01
SM(d17:1/16:0)	1.1675	0.9010	1.5128	2.34E-01	4.64E-01
SM(d18:0/16:0)	1.1835	0.9154	1.5300	1.93E-01	4.21E-01
SM(d18:1/16:0)	1.2720	0.9962	1.6240	5.35E-02	2.20E-01
SM(d18:2/16:0)	1.0643	0.8002	1.4154	6.61E-01	7.91E-01
SM(34:3)	0.8636	0.6402	1.1651	3.29E-01	5.75E-01
SM(d16:1/19:0)	1.1372	0.7689	1.6819	5.11E-01	6.87E-01
SM(d18:1/17:0)/ SM(d17:1/18:0)	1.0889	0.8439	1.4050	5.04E-01	6.87E-01
SM(d18:2/17:0)	0.9857	0.7330	1.3255	9.22E-01	9.67E-01
SM(35:2) (b)	1.2586	0.9382	1.6883	1.22E-01	3.55E-01
SM(d18:1/18:0)/ SM(d16:1/20:0)	1.0552	0.8154	1.3654	6.76E-01	7.97E-01
SM(d18:2/18:0)	0.9342	0.6926	1.2600	6.48E-01	7.88E-01
SM(d18:2/18:1)	0.9525	0.7019	1.2927	7.49E-01	8.63E-01
SM(37:1)	0.8157	0.6042	1.1013	1.78E-01	3.99E-01
SM(37:2)	0.8895	0.6831	1.1582	3.76E-01	6.20E-01
SM(d18:1/20:0)/ SM(d16:1/22:0)	0.8895	0.7098	1.1146	3.01E-01	5.44E-01
SM(d18:2/20:0)	0.8917	0.6840	1.1624	3.88E-01	6.21E-01
SM(38:3) (a)	1.2056	0.8960	1.6221	2.10E-01	4.33E-01
SM(38:3) (b)	0.8289	0.6357	1.0808	1.61E-01	3.99E-01
SM(d16:1/23:0)/ SM(d17:1/22:0)	0.8111	0.6451	1.0198	7.20E-02	2.61E-01
SM(d18:0/22:0)	0.6528	0.4810	0.8861	7.39E-03	9.36E-02
SM(d18:1/22:0)/ SM(d16:1/24:0)	0.8472	0.6576	1.0916	1.94E-01	4.21E-01
SM(d16:1/24:1)	1.2283	0.9366	1.6108	1.33E-01	3.56E-01
SM(d18:2/22:0)	0.8169	0.6232	1.0706	1.39E-01	3.64E-01
SM(40:3) (a)	1.4111	1.0489	1.8983	2.40E-02	1.66E-01
SM(40:3) (b)	1.1132	0.8203	1.5105	4.82E-01	6.86E-01
SM(41:0)	0.8233	0.6478	1.0464	1.09E-01	3.53E-01
SM(41:1) (a)	0.7672	0.5542	1.0619	1.07E-01	3.53E-01
SM(d18:1/23:0)/ SM(d17:1/24:0)	0.8323	0.6534	1.0603	1.33E-01	3.56E-01
SM(d17:1/24:1)	1.3076	0.9977	1.7138	5.19E-02	2.19E-01
SM(d18:2/23:0)	0.7940	0.6173	1.0213	7.15E-02	2.61E-01
SM(d18:1/24:0)	0.8576	0.6464	1.1377	2.79E-01	5.17E-01
SM(d18:1/24:1)	1.4258	1.0608	1.9165	1.99E-02	1.66E-01
SM(d18:2/24:0)	0.8489	0.6427	1.1212	2.41E-01	4.64E-01
SM(43:1)	0.8782	0.6437	1.1981	4.03E-01	6.26E-01
SM(d19:1/24:1)	1.1609	0.7550	1.7849	4.88E-01	6.86E-01
SM(43:2) (a)	1.4216	1.0791	1.8728	1.36E-02	1.38E-01
SM(43:2) (b)	0.9800	0.7236	1.3272	8.94E-01	9.50E-01
SM(44:1)	0.9729	0.7221	1.3108	8.53E-01	9.26E-01
SM(44:2)	1.2917	0.9553	1.7464	9.42E-02	3.18E-01

SM(44:3)	1.6378	1.2040	2.2279	2.38E-03	5.18E-02
Sulfatide (d18:1:/16:0(OH))	0.8907	0.6300	1.2591	5.03E-01	6.87E-01
Sulfatide (d18:1:/16:0)	0.9246	0.6423	1.3310	6.66E-01	7.91E-01
Sulfatide (d18:1:/24:0(OH))	0.7033	0.4894	1.0106	5.67E-02	2.27E-01
Sulfatide (d18:1:/24:0)	1.0031	0.6876	1.4634	9.87E-01	9.94E-01
Sulfatide (d18:1:/24:1(OH))	0.9385	0.6343	1.3888	7.45E-01	8.63E-01
Sulfatide (d18:1:/24:1)	1.3033	0.8900	1.9086	1.68E-01	3.99E-01
**Aged vs Centenarians**					
Cer(d18:0/16:0)	0.8093	0.6575	0.9961	4.61E-02	1.25E-01
Cer(d18:0/18:0)	0.9619	0.6747	1.3715	8.26E-01	9.10E-01
Cer(d18:0/20:0)	0.8527	0.6898	1.0540	1.36E-01	2.69E-01
Cer(d18:0/22:0)	0.5927	0.4884	0.7191	2.81E-06	9.86E-05
Cer(d18:0/24:0)	0.5731	0.4657	0.7053	3.24E-06	9.86E-05
Cer(d18:0/24:1)	1.0991	0.8809	1.3713	3.93E-01	5.64E-01
Cer(d16:1/16:0)	1.0913	0.8586	1.3869	4.66E-01	6.05E-01
Cer(d16:1/18:0)	1.1224	0.8143	1.5473	4.71E-01	6.07E-01
Cer(d16:1/20:0)	0.9931	0.7477	1.3191	9.61E-01	9.68E-01
Cer(d16:1/22:0)	0.7733	0.5542	1.0789	1.27E-01	2.56E-01
Cer(d16:1/23:0)	0.8643	0.6202	1.2046	3.80E-01	5.50E-01
Cer(d16:1/24:0)	0.6538	0.4826	0.8858	7.31E-03	4.11E-02
Cer(d16:1/24:1)	1.4174	1.0355	1.9402	3.03E-02	9.81E-02
Cer(d17:1/16:0)	1.2330	0.9526	1.5958	1.09E-01	2.29E-01
Cer(d17:1/18:0)	1.1636	0.9335	1.4503	1.72E-01	3.23E-01
Cer(d17:1/20:0)	1.3460	0.9984	1.8146	5.12E-02	1.34E-01
Cer(d17:1/22:0)	0.9844	0.7196	1.3467	9.20E-01	9.51E-01
Cer(d17:1/23:0)	1.0266	0.7367	1.4307	8.74E-01	9.30E-01
Cer(d17:1/24:0)	0.8182	0.5966	1.1223	2.07E-01	3.66E-01
Cer(d17:1/24:1)	1.6407	1.1809	2.2795	4.15E-03	3.00E-02
Cer(d18:1/14:0)	1.2618	0.8747	1.8204	2.07E-01	3.66E-01
Cer(d18:1/16:0)	1.3648	1.0630	1.7522	1.60E-02	6.94E-02
Cer(d18:1/18:0)	1.4763	1.0880	2.0031	1.37E-02	6.19E-02
Cer(d18:1/19:0)	1.1168	1.0157	1.2280	2.37E-02	8.38E-02
Cer(d18:1/20:0)	1.4200	1.0784	1.8699	1.39E-02	6.19E-02
Cer(d18:1/21:0)	1.7871	1.3275	2.4059	3.18E-04	4.03E-03
Cer(d18:1/22:0)	0.9589	0.7280	1.2630	7.60E-01	8.68E-01
Cer(d18:1/23:0)	1.0194	0.7638	1.3606	8.93E-01	9.37E-01
Cer(d18:1/24:0)	0.8054	0.6096	1.0640	1.24E-01	2.55E-01
Cer(d18:1/24:1)	1.9270	1.4420	2.5749	4.71E-05	8.95E-04
Cer(d18:1/26:0)	1.0289	0.8003	1.3228	8.20E-01	9.10E-01
Cer(d18:2/14:0)	0.9609	0.5780	1.5974	8.75E-01	9.30E-01
Cer(d18:2/16:0)	1.1707	0.9062	1.5123	2.21E-01	3.78E-01
Cer(d18:2/17:0)	1.1631	1.0280	1.3158	1.77E-02	7.17E-02
Cer(d18:2/18:0)	1.1803	0.8244	1.6899	3.56E-01	5.30E-01
Cer(d18:2/20:0)	1.0212	0.7246	1.4392	9.02E-01	9.39E-01

Cer(d18:2/21:0)	1.4678	1.0187	2.1149	4.00E-02	1.15E-01
Cer(d18:2/22:0)	0.8603	0.5964	1.2411	4.11E-01	5.69E-01
Cer(d18:2/23:0)	0.9602	0.6669	1.3826	8.23E-01	9.10E-01
Cer(d18:2/24:0)	0.7375	0.5219	1.0423	8.28E-02	1.82E-01
Cer(d18:2/24:1)	1.5899	1.1203	2.2564	1.08E-02	5.37E-02
Cer(d18:2/26:0)	0.9370	0.6833	1.2849	6.79E-01	8.02E-01
Cer(d19:1/16:0)	1.0360	0.6726	1.5955	8.69E-01	9.30E-01
Cer(d19:1/18:0)	1.3451	0.8332	2.1713	2.18E-01	3.78E-01
Cer(d19:1/20:0)	1.2076	0.8123	1.7953	3.42E-01	5.14E-01
Cer(d19:1/22:0)	0.8052	0.5316	1.2196	2.98E-01	4.81E-01
Cer(d19:1/23:0)	0.8588	0.5537	1.3320	4.87E-01	6.22E-01
Cer(d19:1/24:0)	0.6836	0.4501	1.0384	7.34E-02	1.77E-01
Cer(d19:1/24:1)	1.4808	0.9490	2.3107	8.21E-02	1.82E-01
Cer(d19:1/26:0)	0.8574	0.6673	1.1015	2.22E-01	3.78E-01
Cer(d20:1/22:0)	0.9248	0.7943	1.0767	3.05E-01	4.83E-01
Cer(d20:1/23:0)	0.7980	0.5884	1.0822	1.42E-01	2.77E-01
Cer(d20:1/24:0)	0.6864	0.5324	0.8850	4.75E-03	3.28E-02
Cer(d20:1/24:1)	1.2963	0.9066	1.8536	1.50E-01	2.89E-01
Cer(d20:1/26:0)	0.8198	0.6439	1.0437	1.04E-01	2.23E-01
Cer1P(d18:1/16:0)	1.2195	0.9487	1.5675	1.18E-01	2.46E-01
GM1(d18:1/16:0)	1.2343	0.9720	1.5674	8.25E-02	1.82E-01
GM3(d18:1/16:0)	1.5861	1.2697	1.9814	1.53E-04	2.16E-03
GM3(d18:1/18:0)	0.9035	0.7000	1.1661	4.26E-01	5.69E-01
GM3(d18:1/20:0)	1.6058	1.2160	2.1205	1.38E-03	1.20E-02
GM3(d18:1/22:0)	1.2226	0.8946	1.6709	2.01E-01	3.66E-01
GM3(d18:1/24:0)	1.0658	0.7915	1.4351	6.67E-01	7.99E-01
GM3(d18:1/24:1)	2.0545	1.5756	2.6791	2.67E-06	9.86E-05
GM3(d18:2/24:1)	1.7419	1.3575	2.2353	5.94E-05	1.00E-03
Hex1Cer(d16:1/18:0)	1.0975	0.8070	1.4925	5.44E-01	6.78E-01
Hex1Cer(d16:1/20:0)	1.1242	0.8372	1.5096	4.27E-01	5.69E-01
Hex1Cer(d16:1/22:0)	0.9706	0.7764	1.2133	7.88E-01	8.87E-01
Hex1Cer(d16:1/24:0)	1.0490	0.8259	1.3325	6.88E-01	8.04E-01
Hex1Cer(d18:1/16:0)	1.5990	1.3090	1.9533	2.79E-05	6.07E-04
Hex1Cer(d18:1/18:0)	1.3021	1.0247	1.6547	3.17E-02	9.89E-02
Hex1Cer(d18:1/20:0)	1.3183	1.0849	1.6019	6.62E-03	4.02E-02
Hex1Cer(d18:1/22:0)	1.0741	0.8581	1.3444	5.23E-01	6.57E-01
Hex1Cer(d18:1/24:0)	1.1264	0.8851	1.4334	3.24E-01	4.98E-01
Hex1Cer(d18:1/24:1)	1.9133	1.5095	2.4252	2.29E-06	9.86E-05
Hex1Cer(d18:2/18:0)	1.1336	0.8879	1.4474	3.06E-01	4.83E-01
Hex1Cer(d18:2/20:0)	1.1083	0.8185	1.5007	4.97E-01	6.29E-01
Hex1Cer(d18:2/22:0)	1.0499	0.8277	1.3317	6.81E-01	8.02E-01
Hex1Cer(d18:2/24:0)	1.1398	0.8820	1.4729	3.08E-01	4.83E-01
Hex2Cer(d16:1/16:0)	1.2350	1.0121	1.5069	3.82E-02	1.12E-01
Hex2Cer(d16:1/24:1)	1.3428	1.0301	1.7503	3.02E-02	9.81E-02
Hex2Cer(d18:1/16:0)	1.1645	0.9178	1.4776	2.03E-01	3.66E-01

Hex2Cer(d18:1/18:0)	1.0663	0.8184	1.3894	6.26E-01	7.55E-01
Hex2Cer(d18:1/20:0)	1.1497	0.8935	1.4793	2.70E-01	4.46E-01
Hex2Cer(d18:1/22:0)	1.1025	0.8449	1.4387	4.63E-01	6.05E-01
Hex2Cer(d18:1/24:0)	1.0472	0.8175	1.3413	7.08E-01	8.22E-01
Hex2Cer(d18:1/24:1)	1.4899	1.1231	1.9764	6.88E-03	4.02E-02
Hex2Cer(d18:2/16:0)	1.2656	0.9944	1.6109	5.53E-02	1.43E-01
Hex2Cer(d18:2/24:1)	1.4152	1.0604	1.8887	1.96E-02	7.34E-02
Hex3Cer(d18:1/16:0)	1.5053	1.2107	1.8716	4.98E-04	5.40E-03
Hex3Cer(d18:1/18:0)	1.4910	1.1494	1.9341	3.53E-03	2.69E-02
Hex3Cer(d18:1/20:0)	1.5417	1.1940	1.9908	1.46E-03	1.20E-02
Hex3Cer(d18:1/22:0)	1.3285	1.0530	1.6761	1.79E-02	7.17E-02
Hex3Cer(d18:1/24:0)	1.3304	1.0316	1.7156	2.88E-02	9.72E-02
Hex3Cer(d18:1/24:1)	2.1141	1.6965	2.6346	3.13E-08	4.76E-06
S1P(d16:1)	0.9105	0.7201	1.1513	4.24E-01	5.69E-01
S1P(d17:1)	1.1456	0.8727	1.5039	3.19E-01	4.94E-01
S1P(d18:0)	0.8862	0.6655	1.1801	3.99E-01	5.66E-01
S1P(d18:1)	1.0366	0.8114	1.3243	7.68E-01	8.71E-01
S1P(d18:2)	0.8908	0.6717	1.1815	4.13E-01	5.69E-01
Sph(d16:1)	0.9771	0.8486	1.1249	7.41E-01	8.53E-01
Sph(d18:1)	0.9546	0.8155	1.1173	5.54E-01	6.84E-01
Sph(d18:2)	0.9851	0.8338	1.1640	8.57E-01	9.30E-01
SM(d17:1/14:0)	1.1245	0.8692	1.4548	3.63E-01	5.35E-01
SM(d18:0/14:0)	1.0639	0.8513	1.3297	5.77E-01	7.08E-01
SM(d18:1/14:0)/ SM(d16:1/16:0)	1.0862	0.8799	1.3409	4.32E-01	5.71E-01
SM(d18:2/14:0)	1.1256	0.8789	1.4417	3.39E-01	5.14E-01
SM(d17:1/16:0)	1.2409	0.9906	1.5545	5.99E-02	1.49E-01
SM(d18:0/16:0)	1.1931	0.9710	1.4659	9.08E-02	1.97E-01
SM(d18:1/16:0)	1.2321	1.0025	1.5144	4.75E-02	1.27E-01
SM(d18:2/16:0)	1.1889	0.9274	1.5241	1.67E-01	3.17E-01
SM(34:3)	0.9001	0.6905	1.1733	4.27E-01	5.69E-01
SM(d16:1/19:0)	0.9863	0.7010	1.3877	9.35E-01	9.57E-01
SM(d18:1/17:0)/ SM(d17:1/18:0)	1.1549	0.9286	1.4362	1.89E-01	3.51E-01
SM(d18:2/17:0)	1.1103	0.8616	1.4307	4.09E-01	5.69E-01
SM(35:2) (b)	1.2689	0.9747	1.6518	7.55E-02	1.79E-01
SM(d18:1/18:0)/ SM(d16:1/20:0)	1.0086	0.8088	1.2578	9.38E-01	9.57E-01
SM(d18:2/18:0)	1.0013	0.7741	1.2952	9.92E-01	9.92E-01
SM(d18:2/18:1)	0.9332	0.7187	1.2118	5.95E-01	7.24E-01
SM(37:1)	0.5309	0.4081	0.6906	1.88E-05	4.77E-04
SM(37:2)	0.8109	0.6442	1.0208	7.31E-02	1.77E-01
SM(d18:1/20:0)/ SM(d16:1/22:0)	0.8077	0.6624	0.9849	3.55E-02	1.06E-01
SM(d18:2/20:0)	0.8710	0.6936	1.0937	2.27E-01	3.80E-01
SM(38:3) (a)	1.1478	0.8828	1.4924	2.95E-01	4.81E-01
SM(38:3) (b)	0.6962	0.5405	0.8966	6.18E-03	3.95E-02

SM(d16:1/23:0)/ SM(d17:1/22:0)	0.7852	0.6303	0.9782	3.19E-02	9.89E-02
SM(d18:0/22:0)	0.6143	0.4758	0.7931	4.17E-04	4.88E-03
SM(d18:1/22:0)/ SM(d16:1/24:0)	0.7400	0.5954	0.9198	7.90E-03	4.17E-02
SM(d16:1/24:1)	1.2518	1.0073	1.5557	4.31E-02	1.19E-01
SM(d18:2/22:0)	0.7482	0.5877	0.9525	1.98E-02	7.34E-02
SM(40:3) (a)	1.3764	1.0637	1.7810	1.64E-02	6.94E-02
SM(40:3) (b)	1.0063	0.7756	1.3055	9.61E-01	9.68E-01
SM(41:0)	0.8071	0.6627	0.9828	3.38E-02	1.03E-01
SM(41:1) (a)	0.6598	0.4933	0.8825	6.23E-03	3.95E-02
SM(d18:1/23:0)/ SM(d17:1/24:0)	0.8102	0.6521	1.0066	5.70E-02	1.44E-01
SM(d17:1/24:1)	1.4446	1.1619	1.7961	1.50E-03	1.20E-02
SM(d18:2/23:0)	0.7874	0.6264	0.9897	4.09E-02	1.15E-01
SM(d18:1/24:0)	0.7317	0.5775	0.9269	1.10E-02	5.37E-02
SM(d18:1/24:1)	1.5257	1.2092	1.9250	7.12E-04	7.22E-03
SM(d18:2/24:0)	0.7418	0.5800	0.9488	1.87E-02	7.27E-02
SM(43:1)	0.8257	0.6436	1.0594	1.28E-01	2.56E-01
SM(d19:1/24:1)	1.1706	0.8208	1.6694	3.75E-01	5.48E-01
SM(43:2) (a)	1.5287	1.2452	1.8767	1.57E-04	2.16E-03
SM(43:2) (b)	0.9842	0.7776	1.2456	8.92E-01	9.37E-01
SM(44:1)	0.8680	0.6885	1.0944	2.24E-01	3.78E-01
SM(44:2)	1.2884	1.0297	1.6121	2.77E-02	9.58E-02
SM(44:3)	1.5665	1.2110	2.0264	1.09E-03	1.04E-02
Sulfatide (d18:1:/16:0(OH))	0.6534	0.4700	0.9085	1.27E-02	6.04E-02
Sulfatide (d18:1:/16:0)	0.7314	0.5166	1.0354	7.64E-02	1.79E-01
Sulfatide (d18:1:/24:0(OH))	0.6302	0.4514	0.8800	7.96E-03	4.17E-02
Sulfatide (d18:1:/24:0)	0.7228	0.5028	1.0390	7.81E-02	1.80E-01
Sulfatide (d18:1:/24:1(OH))	0.6676	0.4739	0.9403	2.20E-02	7.96E-02
Sulfatide (d18:1:/24:1)	1.0327	0.7325	1.4561	8.51E-01	9.30E-01

Supplementary Table 4. Sphingolipid species C24:0/C24:1 ratio in adults, aged and centenarians groups.

C24:0/24:1 ratio	Adults	Aged	Centenarian
Hex1Cer(d18:1/24:0):Hex1Cer(d18:1/24:1)	1.6050 ± 0.3077	1.4863 ± 0.4100	0.9498 ± 0.2825
Hex2Cer(d18:1/24:0):Hex2Cer(d18:1/24:1)	0.5154 ± 0.2094	0.5325 ± 0.2094	0.3695 ± 0.1087
Hex3Cer(d18:1/24:0):Hex3Cer(d18:1/24:1)	0.4967 ± 0.1001	0.4595 ± 0.1117	0.3163 ± 0.0862
Cer(d16:1/24:0):Cer(d16:1/24:1)	4.2662 ± 1.1538	3.4303 ± 1.0717	1.9806 ± 0.5976
Cer(d17:1/24:0):Cer(d17:1/24:1)	2.5874 ± 0.5107	2.0347 ± 0.5569	1.2738 ± 0.2864
Cer(d18:0/24:0):(d18:0/24:1)	1.9619 ± 0.5495	1.5490 ± 0.4515	1.0227 ± 0.2568

Cer(d18:1/24:0):(d18:1/24:1)	3.2315 ± 0.8389	2.5492 ± 0.8053	1.3500 ± 0.3695
Cer(d18:2/24:0):(d18:2/24:1)	4.5371 ± 1.1295	3.7891 ± 1.1607	2.1019 ± 0.6129
Cer(d20:1/24:0):(d20:1/24:1)	2.7765 ± 0.9520	2.2631 ± 0.7154	1.4515 ± 0.4054 *

*, $p<0.05$
